# The first oviraptorosaur (Dinosauria: Theropoda) bonebed: evidence of gregarious behaviour in a maniraptoran theropod

**DOI:** 10.1038/srep35782

**Published:** 2016-10-21

**Authors:** Gregory F. Funston, Philip J. Currie, David A. Eberth, Michael J. Ryan, Tsogtbaatar Chinzorig, Demchig Badamgarav, Nicholas R. Longrich

**Affiliations:** 1 University of Alberta, Department of Biological Sciences, CW405 Biological Sciences Building, Edmonton, Alberta, T6G 2E9, Canada; 2 Royal Tyrrell Museum of Palaeontology, Box 7500, Drumheller, Alberta, T0J 0Y0 Canada; 3 Department of Vertebrate Paleontology, Cleveland Museum of Natural History, 1 Wade Oval Dr., University Circle, Cleveland, OH 44106, USA; 4 Hokkaido University Museum, Hokkaido University, Sapporo 060-0810, Japan; 5 Paleontological Center, Mongolian Academy of Sciences, Box 260, Ulaan Baatar 210351, Mongolia; 6 Department of Biology and Biochemistry, University of Bath, Claverton Down, Bath, United Kingdom

## Abstract

A monodominant bonebed of *Avimimus* from the Nemegt Formation of Mongolia is the first oviraptorosaur bonebed described and the only recorded maniraptoran bonebed from the Late Cretaceous. Cranial elements recovered from the bonebed provide insights on the anatomy of the facial region, which was formerly unknown in *Avimimus*. Both adult and subadult material was recovered from the bonebed, but small juveniles are underrepresented. The taphonomic and sedimentological evidence suggests that the *Avimimus* bonebed represents a perimortem gregarious assemblage. The near absence of juveniles in the bonebed may be evidence of a transient age-segregated herd or ‘flock’, but the behaviour responsible for this assemblage is unclear. Regardless, the *Avimimus* bonebed is the first evidence of gregarious behaviour in oviraptorosaurs, and highlights a potential trend of increasing gregariousness in dinosaurs towards the end of the Mesozoic.

Dinosaur sociality—as inferred from the close association of multiple, putatively related individuals—is now supported in many clades by numerous different lines of evidence, including trackways^1^, nesting sites^2^, and mass death assemblages (bonebeds). Extant birds, many of which have complex social behaviours, are now recognized as modified maniraptoran dinosaurs^3^,^4^,^5^; however, the origins of these behaviours in theropods is currently poorly understood.

Monodominant bonebeds are often mass death assemblages dominated by single taxa. Such deposits are relatively common for dinosaurs throughout the Mesozoic, especially the Cretaceous period. These bonebeds are typically interpreted as reflecting natural perimortem associations of the preserved taxa^6^,^7^,^8^,^9^,^10^,^11^, rather than postmortem abiotic aggregations, and thus are evidence of gregariousness or sociality. During the Late Cretaceous, certain groups, including ankylosaurs^12^,^13^,^14^, ceratopsians^8^,^15^,^16^, hadrosaurs^9^,^10^, ornithomimids^17^,^18^, and tyrannosaurs^19^, show strong tendencies to form monodominant assemblages, regardless of disparate preservation potentials and paleoenvironmental preferences. Some other Late Cretaceous clades, however, especially those on the stem lineage of birds, are unknown from bonebeds despite comparable fossil records. One such group, oviraptorosaurs, share remarkable convergences with birds^20^,^21^,^22^ and are important for understanding the changes that occurred during the dinosaur-bird transition^22^.

One of the most birdlike oviraptorosaurs is *Avimimus*, first described on the basis of an associated skeleton from Udan Sayr^20^, but also known from other Upper Cretaceous Mongolian localities including Bugin Tsav^23^, Khermeen Tsav^23^, Shar Tsav^20^,^24^,^25^, and Yagaan Khovil^24^. *Avimimus* is characterized by many unusually birdlike features, including edentulous jaws, a prominent antitrochanter of the ilium, and fusion of various skeletal elements to create compound bones, including a neurocranium, carpometacarpus, synsacrum, tibiotarsus, and tarsometatarsus^20^,^26^,^27^,^28^,^29^. Kurzanov^20^ reconstructed *Avimimus* with a well-developed complement of feathers, a conclusion that was prescient given the discovery of large remiges and rectrices in the basal oviraptorosaurs *Caudipteryx* and *Protarchaeopteryx*^30^.

In 2006, a poached *Avimimus* bonebed (Figs 1 and 2) was discovered at the Nemegt locality in Mongolia^31^. When discovered, poachers had excavated the bottom of a low, isolated hill, and broken small theropod bones were found on and within the spoil piles around the quarry. The expedition members recognized that the broken bones represent multiple individuals of the relatively rare *Avimimus*, so a systematic excavation was initiated at the back of the existing quarry, where undisturbed material was found. The excavation was extended by 2007 and 2016 expeditions, which uncovered the remains of additional individuals of *Avimimus*, now housed at the Institute of Paleontology and Geology, Mongolian Academy of Sciences (MPC-D) in Ulaanbaatar, Mongolia. The assemblage is the first reported record of a monodominant bonebed of oviraptorosaurs—as well as the first Late Cretaceous maniraptoran bonebed—and provides insights on the anatomy and behaviour of *Avimimus*.

## Results

### Geological and Sedimentological Context

**Figure 1:**
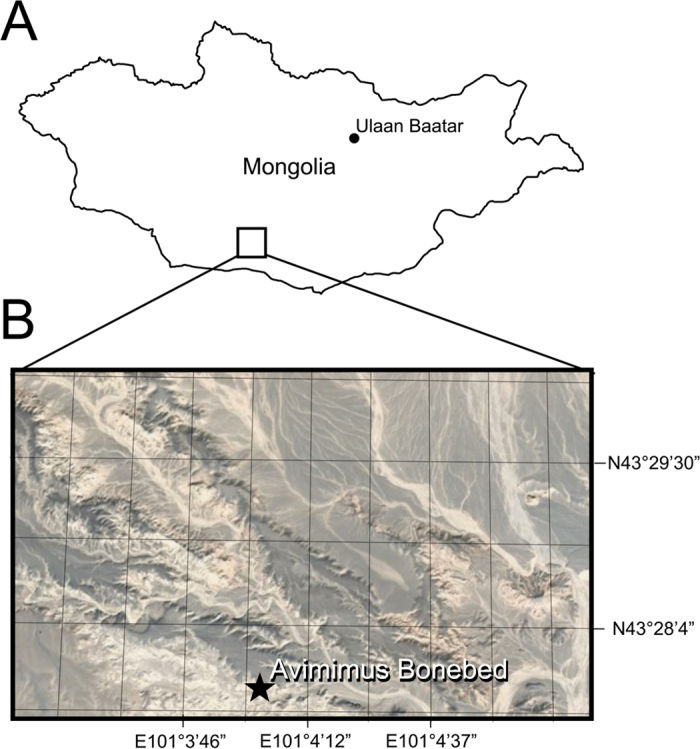
Locality of *Avimimus* bonebed. Map (**A**) of Mongolia traced in Photoshop CS6 (www.adobe.com/photoshop), indicating the region of the Nemegt Locality (**B**). Detail (**B**) of the Nemegt locality, indicating location of *Avimimus* bonebed (pointer). Map data in (**A**) and satellite imagery in (**B**) from Google Maps (Map data: © Google), used under fair use terms.

The *Avimimus* bonebed occurs in the lower portion of the alluvial Nemegt Formation, at the Nemegt locality within the Nemegt Basin^32^,^33^ (Fig. 1). Here, Upper Cretaceous strata of the Nemegt Formation interfinger with and overlie the Baruungoyot Formation^33^. In general the Nemegt Formation is characterized by abundant deposits of ephemerally active meandering channels, splays and sheetfloods, and ponds and wetlands^32^,^33^. The Nemegt Formation has yielded few bonebed deposits; the only two recorded instances include an assemblage of *Saurolophus*^34^ and this *Avimimus* bonebed. The precise age of the Nemegt Formation is difficult to determine because of the discontinuity of beds and exposures, absence of microfossil biostratigraphy, and lack of datable volcanics^33^. Nonetheless, based on the better-documented late Campanian to early Maastrichtian age of the underlying Djadokhta Formation^35^ and the presence of Maastrichtian dinosaurs such as *Saurolophus* in the Nemegt Formation, an early Maastrichtian age for the Nemegt Formation is currently accepted.

**Figure 2:**
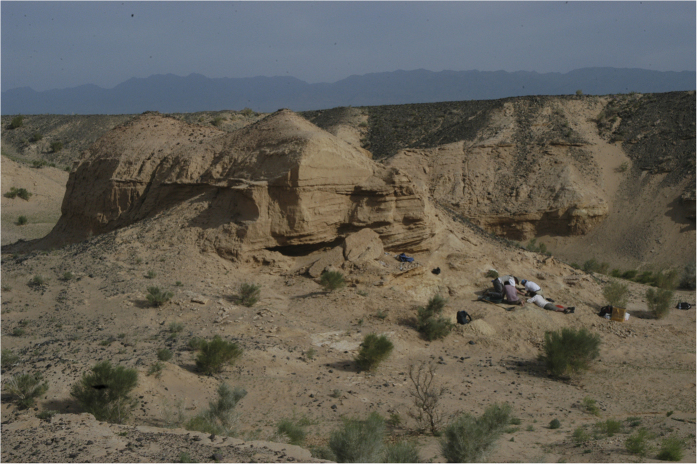
Image of field locality of *Avimimus* bonebed, showing regional sedimentological structures. Note people bottom right for scale.

The exposed Nemegt Formation around the bonebed is only 35 m thick due to truncation by a regionally-expressed Quaternary unconformity. The *Avimimus* bonebed occurs 10.5 m above an interfingered Nemegt-Baruungoyot contact and is associated with the lower portions of sigmoidal and offlapping inclined beds of silty, pebbly, fine- to medium-grained sandstone^33^ (Fig. 2). Two types of matrix surround the bonebed. The base of the bonebed is a fine-grained sandstone, which is overlain by a coarse-grained sandstone with some clay rip-up clasts. Inclined beds are typically less than 5 cm thick, exhibit a total vertical relief of 40 cm, and dip toward the south and southwest, suggesting that they are offlapping deposits of a migrating point bar in a meandering river channel^32^,^33^. Large-scale trough cross beds drape the toes of the point bar and immediately overlie the bonebed. Paleocurrent data collected from them indicate that flow at the base of the point-bar was toward the west-southwest, ranging from 240–280°. Bones are preserved in both matrix types, and some bones span the contact between the layers. This suggests that the beds were deposited in a single coarsening-upwards event, probably tied to the migration of the point bar. The presence of localized mudstone pebbles and *Avimimus* remains at the base of the beds, as well as non-predictable grain size changes between beds, poorly organized mixtures of trough and ripple cross-strata within beds, soft-sediment deformation structures, and localized millimeter-thick clay drapes all suggest highly variable conditions through time at the bonebed site. These conditions include erosive hydraulic flow, standing water, subaerial exposure, and trampling by dinosaurs. Accordingly, at times this channel-hosted site may also have acted as a waterhole, attracting vertebrates in search of food and/or water.

### Assemblage

The bonebed assemblage is dominated by *Avimimus*, which comprises 160 (90.4%) of the 177 accessioned specimens (Table S1). The other 17 accessioned specimens include indeterminate dinosaur elements (6), an oviraptorid dorsal centrum, an oviraptorid ilium, embryonic hadrosaur bones, a bird tarsometatarsus, a lizard vertebra, a mammalian limb bone, eggshell, gastropod and bivalve casts, and wood. The relative abundance and dominance of *Avimimus* in the bonebed is underestimated because, in some cases, multiple elements are preserved in articulation or as fused single functional elements that were accessioned together. The non-poached, newly excavated part of the bonebed was collected exhaustively, so it is unlikely that there was a collection bias towards *Avimimus* at the expense of other taxa. *Avimimus* material collected includes a variety of cranial elements, vertebrae, forelimb material, some parts of the pelvis, and many hindlimb elements. Ontogenetic stage of elements from the bonebed was assessed using size and fusion of the elements. Although these qualities are not strictly tied to developmental age, similar criteria have been used previously to assess relative age in other assemblages where histological sections were unavailable^8^,^36^. Adults were identified by fusion of the tibiotarsus or tarsometatarsus. The lengths of fused tibiatarsi (n = 17) from the bonebed vary by less than 10% (246 mm – 280 mm), suggesting that adults were either all of a similar ontogenetic stage, or that growth was determinate in adult individuals. All tibiotarsi longer than 246 mm (n = 17) are fused, and none shorter than 246 mm are fused, indicating that fusion of the tibiotarsus is strongly tied to body size. Subadult individuals were identified by lack of hindlimb fusion but proximity in size to the largest fused individuals (tibiae >80% of 280 mm). Of the 33 tibiae recovered from the bonebed (Table 1), only one tibia (MPC-D 102/38), an estimated 202 mm in length, fell below the 80% cutoff of the length of the largest tibia (280 mm), and can be considered a juvenile individual. The state of fusion for this individual cannot be assessed, because the distal end is missing. The presence in the assemblage of small elements from *Avimimus*, such as phalanges, and small material from other taxa, including embryonic hadrosaur bones, a lizard vertebra, and mammal limb bones, suggests that the dearth of juvenile *Avimimus* is real, rather than the result of winnowing. Evidence from other bonebeds suggests that non-avian theropod dinosaurs had a tendency to form juvenile-dominated herds^17^,^36^,^37^,^38^. Adult-dominated bonebeds of non-theropod taxa also typically contain a small percentage of juvenile animals^8^,^34^. The *Avimimus* bonebed is therefore unusual in its near absence of juvenile individuals.

The minimum number of individuals (MNI) represented by the assemblage was estimated using tibiae. The distal ends of 13 right tibiae are present and indicate that at least this many individuals contributed to the assemblage. However, combining the measurements of these 13 specimens with data from left tibiae (Table 1) shows that there are at least 18 tibiae of different sizes in the assemblage. Thus, an MNI of 18 appears to be the best estimate for the number of individuals that contributed to the assemblage, which represents the largest monodominant assemblage of maniraptorans yet reported.

### Taphonomy

**Figure 3:**
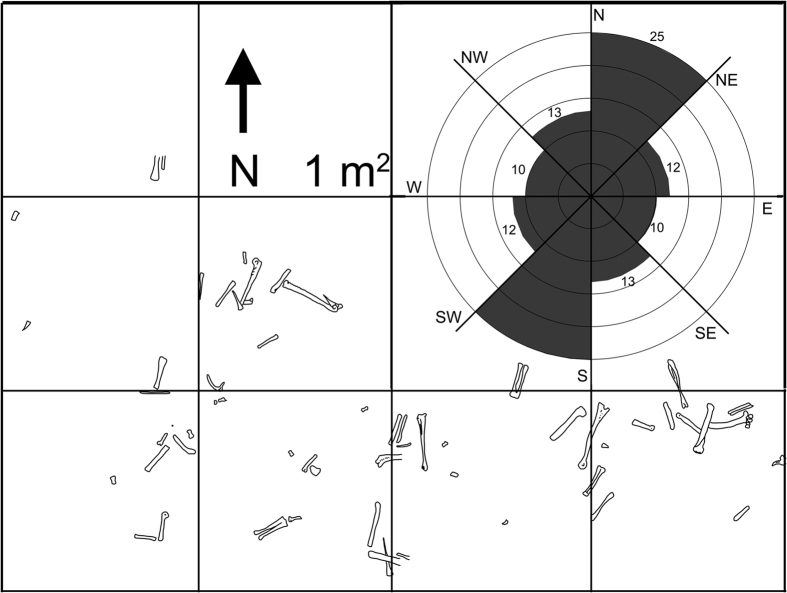
Map of *Avimimus* bonebed, showing orientation and position of hindlimb bones *in situ*. Each square represents 1 m^2^. Arrow indicates North. Mirrored rose diagram indicates orientations of *in situ* long bones in *Avimimus* bonebed. Shaded areas represent the number of bones within each range of orientations (n = 60). Orientation measured from long axis of bones.

**Figure 4:**
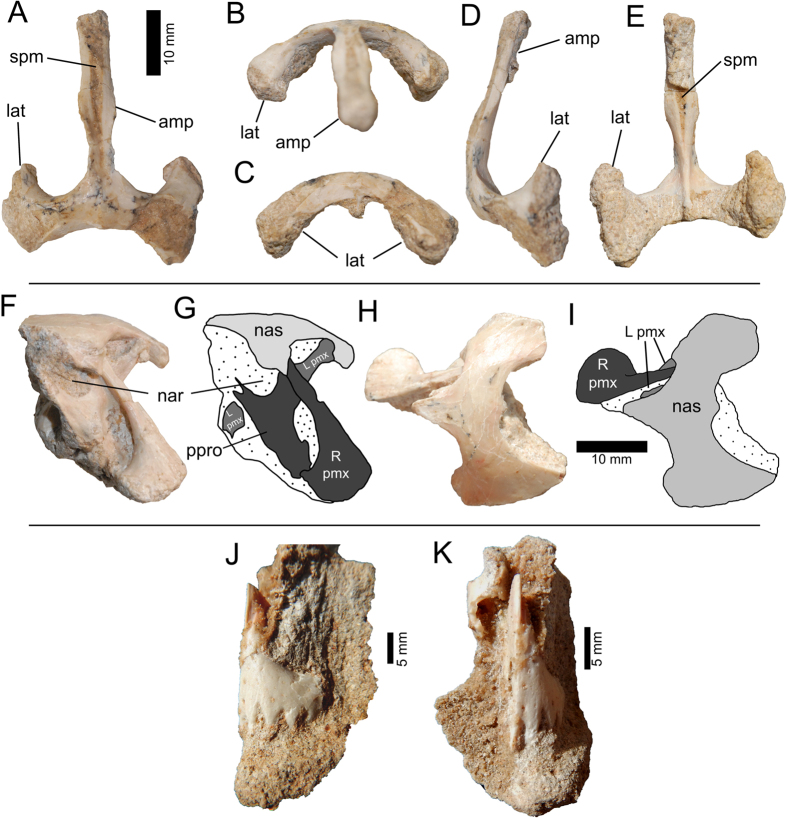
MPC-D 102/46, *Avimimus*. Fused nasals in dorsal (**A**), anterior (**B**), posterior (**C**), right lateral (**D**), and ventral (**E**) views. MPC-D 102/34, *Avimimus*. Block containing right premaxilla, maxilla and fused nasals in lateral (**F**,**G**) and dorsal (**H**,**I**) views. MPC-D 102/108, *Avimimus*. Block containing two nearly articulated premaxillae in left lateral (**J**) and anterior (**K**) views. Abbrevations: **amp**, anterior midline process; **lat**, lateral descending process; **L pmx**, left premaxilla; **nar**, naris; **nas**, nasal; **ppro**, posterior process of premaxilla; **R pmx**, right premaxilla; **spm**, slot in nasal for premaxilla.

Given an MNI of 18, overall skeletal element representation of *Avimimus* is low in the bonebed (~4–5%, Table S2). Hindlimb elements are strongly overrepresented (~30–94%) in the assemblage compared to all other elements (Fig. S1). Long bones excavated from the quarry (Fig. 3) show a significantly preferred NE-SW orientation (Rao t = 201, p < 0.001; Raleigh Z: t = 0.7211, p < 0.001), subparallel to sedimentological indications of a SW-oriented paleochannel. All *Avimimus* bones recovered from the site share the same bone modification signature, lacking signs of prolonged subaerial exposure, insect feeding traces, tooth marks, or weathering. Small fragments of hadrosaur bones in the bonebed are typically abraded, weathered, and powdery, suggesting they have been subject to different taphonomic conditions. Most of the bones were preserved horizontally, but in the coarse-grained sandstone layer several elements (ilium, humerus, phalanges) were vertical or inclined, suggesting that they were moved from resting position and buried quickly. Few of the unfused elements are preserved in articulation, with the notable exceptions of two nearly articulated premaxillae (Fig. 4J,K) and eight distal caudals (Fig. S2) found articulated. Most of the associated material comes from compound or fused elements, but the presence of some associated material (ilium and unfused sacral ribs) indicates that the bones were not transported far. The bones have been hydraulically sorted, so that small elements are rare—but still present—and there is a bias towards the preservation of thick-walled elements like femora and tibiae. Despite this, the bones show little to no abrasion, and delicate elements, like cranial material and fibulae (Fig. S3), are unbroken, which suggests that hydraulic flows that sorted or reoriented elements were not intense or prolonged. Most of the broken bones from the assemblage were surface collected, suggesting that they were damaged by the poachers. Only two theropod teeth (cf. *Velociraptor*) were recovered from the site, suggesting that scavenging, if present at all, was limited. It is unlikely for a number of reasons that the sediments surrounding the bonebed represent the first burial of the material. First, such an assemblage would be dominated by articulated or associated material, rather than isolated bones. Second, the matrix is representative of normal deposition in a channel, rather than a catastrophic flood capable of killing multiple *Avimimus.* The pristine condition of the bones suggests they were originally buried rapidly, which protected them from subaerial weathering, trampling and scavenging, but allowed most of the soft tissues to decompose over the course of months to years. The bonebed was then uncovered by a medium- to high-energy flow, represented by the two sandstones, which disarticulated most of the material and transported it a short distance. The second flow event had enough energy to reorient most of the long bones to a N-NE to S-SW orientation (Fig. 3), but was not powerful enough to remove large elements. Numerous examples of similar multistage depositional events in monodominant assemblages are known from North America^10^,^39^,^40^.

### Craniomandibular Skeleton

The bonebed produced cranial elements that were formerly unknown for *Avimimus* and provide important anatomical information. A jumble of associated bones (MPC-D 102/34) includes the premaxillae and nasals (Fig. 4), and an additional two semiarticulated premaxillae (MPC-D 102/108) were recovered (Fig. 4J,K). The unfused premaxillae are hollow; each has a long dorsal process with a lateral facet for the nasal and a flat medial surface for the adjoining premaxilla. The tomial margin has five denticulations (Fig. 4J). Uniquely amongst oviraptorosaurs, the laterally flaring posterior process (Fig. 4F,G) that separates the maxilla from the external naris has a deep depression, probably confluent with the antorbital fossa. Although Kurzanov^20^ reconstructed *Avimimus* with a conjoined naris and antorbital fenestra, the presence of the posterior process, which is missing in the holotype, indicates that it would have separated the naris and antorbital fenestra, as in all other theropods. The fused nasals (MPC-D 102/46; Fig. 4) form an unusual anchor-shaped bone. Posteriorly the nasals have ventrolaterally extending, hatchet-shaped lateral descending processes. Anteriorly there is a longitudinal groove on the dorsal surface, which opens into a slot for the premaxillae (Fig. 4). The posterior margin of the fused nasals is concave in dorsal view, and the nasals would have been largely separated posteriorly by the frontals. There is a longitudinal ridge on the ventral side of the midline process of the nasals. The united nasals have a smooth exterior surface and lack the pneumatic pitting that is present in oviraptorids^41^,^42^,^43^. Rearticulating the premaxillae and nasals shows that snout was short, with a vertical anterior margin and anteriorly facing nares.

**Figure 5:**
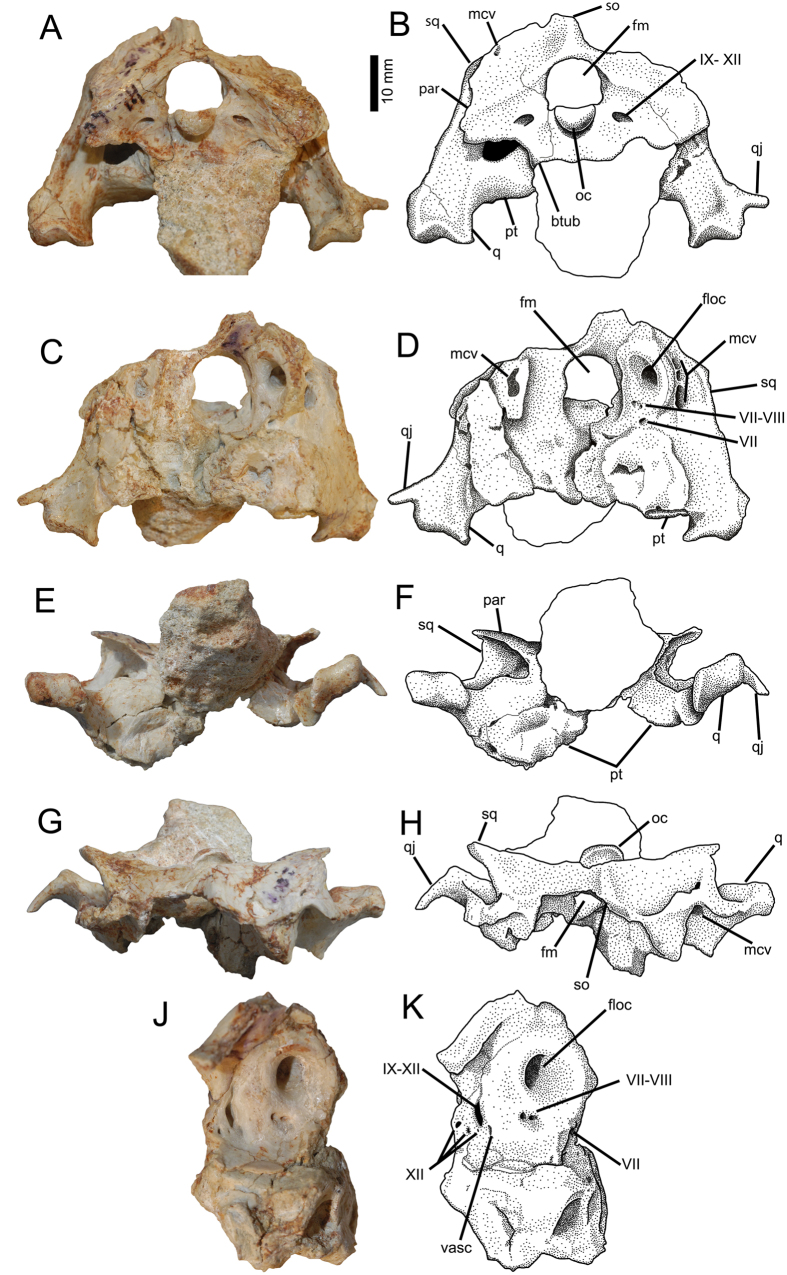
MPC-D 102/81, *Avimimus.* Partial braincase in posterior (**A**,**B**), anterior (**C**,**D**), ventral (**E**,**F**) and dorsal (**G**,**H**) views. Detail of medial wall of exoccipital (**J**,**K**), not to scale. Abbreviations: **btub**, basal tubera; **floc**, floccular fossa; **fm**, foramen magnum; **mcv**, foramina for middle cerebral vein; **oc**, occipital condyle; **pt**, pterygoid; **q**, quadrate; **qj**, quadratojugal; **so**, supraoccipital; **sq**, squamosal; **vasc**, vascular foramen; **VII**, foramen for facial nerve; **VII-VIII**, foramina for branches of the facial nerve and vestibulocochlear nerve; **IX-XII**, foramen for glossopharyngeal, vagus, accessory, and hypoglossal nerves; **XII**, foramina for hypoglossal nerve.

A partial skull (MPC-D 102/81) preserves the posterior part of the cranium, which has coossified into a single unit—here called the neurocranial unit—as in birds. The coossified unit of *Avimimus* incorporates more bones than that of any bird, including the frontals, parietals, postorbitals, pterygoids, quadrates, squamosals, and bones of the braincase. Sutures between bones are obliterated, except for faint lines between the opisthotic-exoccipital unit and the basioccipital. The body of the apneumatic quadrate (Fig. 5) fuses along its whole medial margin to the prootic and pterygoid, so that the only communication of the post-temporal fenestra with the region anterior to the quadrate is the foramen for the middle cerebral vein (Fig. 5C,D). Dorsally, the pterygoid wing of the quadrate and the fused squamosal are separated from the exoccipital by an anteriorly-facing recess with a large ventral foramen and a smaller dorsal fossa, both for the middle cerebral vein. An aperture in the dorsal part of the exoccipital connects this dorsal fossa to the top of the skull. The quadrate condyles are saddle-shaped as in most oviraptorosaurs, suggesting propalinal movement of the mandible was possible^44^. The right quadratojugal is an anteriorly directed prong that is indistinguishably fused to the lateral margin of the quadrate. There is no evidence of a quadratic foramen or fenestra between the quadrate and quadratojugal, a feature that is usually present in oviraptorosaurs^41^,^42^. The occipital process of the squamosal (Fig. 5) is conjoined and fused to the paroccipital process of the exoccipital, which is unusual for oviraptorosaurs. The posterior part of the pterygoid (Fig. 5) is relatively large and is horizontal, contrasting with the typical dorsomedial-ventrolateral orientation of the oviraptorid pterygoid. The pterygoid contacts the basisphenoid along most of its length, rather than just at the basipterygoid process. The pterygoid contact with the quadrate is anteroposteriorly extensive and lies far dorsal to the mandibular condyles of the quadrate. In oviraptorids, the pterygoid typically contacts the quadrate just medial to the mandibular condyle^41^. The broken pterygoid ramus is hollow, unique for oviraptorosaurs (Fig. 5). The occipital condyle (Fig. 5) is kidney-shaped and smaller than the foramen magnum, which is nearly circular as in oviraptorids^43^. The basal tubera are large and separated by a shallow median depression, with a possibly pneumatic foramen at its center. There are no basisphenoid recesses. The basipterygoid processes face laterally and are continuous with the greatly expanded posterior wing of the pterygoid, unlike in oviraptorids^41^. The supraoccipital has a longitudinal sagittal crest, but lacks a transverse nuchal crest. The opisthotic-exoccipitals form small laterally directed paroccipital processes that do not extend ventrally to the level of the basal tubera (Fig. 5). There is only one jugular opening on the posterior surface of each opisthotic-exoccipital—apparently unique for a dinosaur—that served as the exit for cranial nerves IX-XII (Fig. 5). On the medial wall of the exoccipital portion of the braincase (Fig. 5J,K), there are five foramina. The largest of these, dorsal to the others, is a dorsoventrally oriented slit for cranial nerves IX-XI and communicates with the jugular opening on the posterior side of the exoccipital. Of the four smaller, ventral foramina, the anterior one is probably for a blood vessel, and the other three were for branches of cranial nerve XII. The last three foramina merge posteriorly to exit through the large jugular opening on the posterior face of the exoccipital. The medial surface of the braincase is pierced by a large floccular recess, ventral to which is a shallow depression pierced by cranial nerves VII and VIII (Figs 5b and 7J,K). Anterolaterally, the prootic is pierced by a small anteriorly-facing foramen for cranial nerve VII.

**Figure 6:**
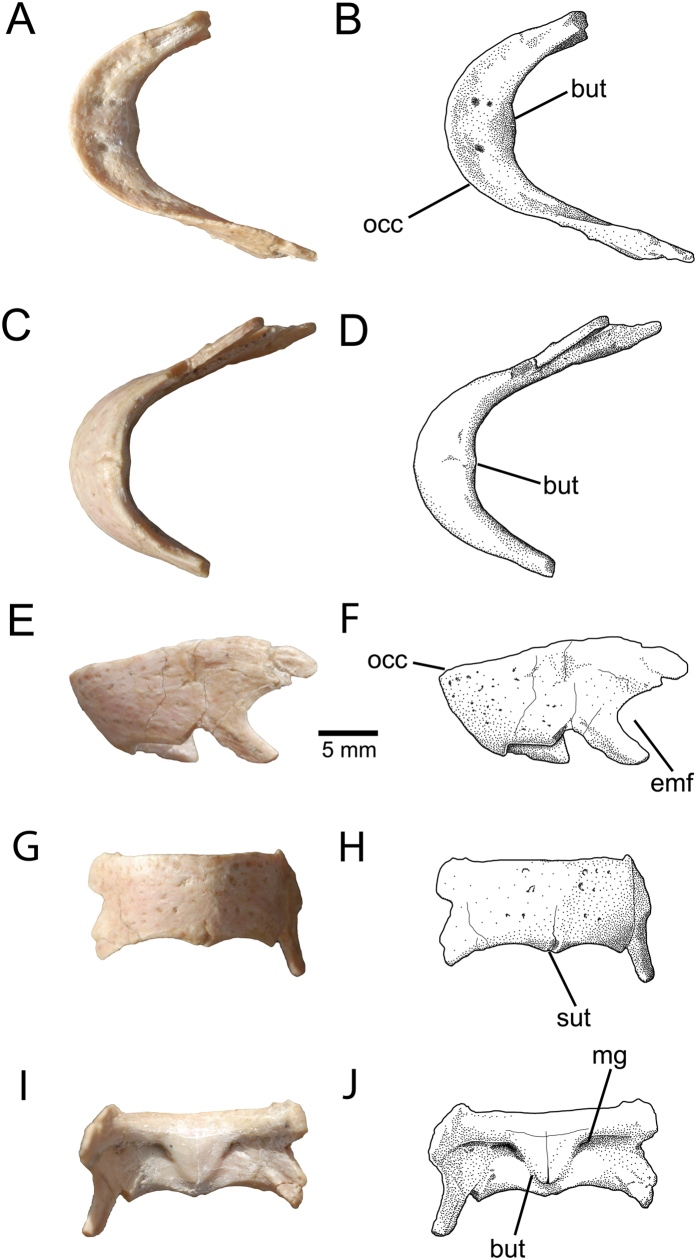
MPC-D 102/16, *Avimimus*. Partial dentaries in dorsal (**A**,**B**), ventral (**C**,**D**), anterior (**E**,**F**), left lateral (**G**,**H**), and posterior (**I**,**J**) views. Abbreviations: **but**, symphyseal buttress; **emf**, external mandibular foramen; **for**, vascular foramen; **lg**, lingual groove; **lr**, lingual ridge; **mg**, Meckelian groove; **occ**, occlusal margin, **sut**, symphyseal suture.

The edentulous apneumatic dentaries (MPC-D 102/16) of *Avimimus* are partly coossified, although a suture is visible ventrally (Fig. 6). The lingual surface of the dentary has a complex series of ridges and grooves, although the relief is not as great as in caenagnathids^44^. There is a distinct lingual groove on the occlusal surface of the dentary, which is bounded medially by a weakly pronounced lingual ridge. There is an incipient symphysial shelf, similar in development to most oviraptorids^41^.The occlusal margin projects above the rest of the lingual surface, but is not concave in lateral view (Fig. 6). The Meckelian grooves are separated at the midline by a distinctive ventrally tapering buttress of bone (Fig. 6), which demarcates two shallow lateral fossae in posterior view. The lateral surface of the mandible is marked by several minute foramina, which suggests that there was a keratinous beak as in birds^44^. The posterodorsal ramus of the dentary is not bifurcated transversely, which indicates that its contact with the surangular or coronoid was simple, as in oviraptorids^41^.

## Discussion

**Figure 7:**
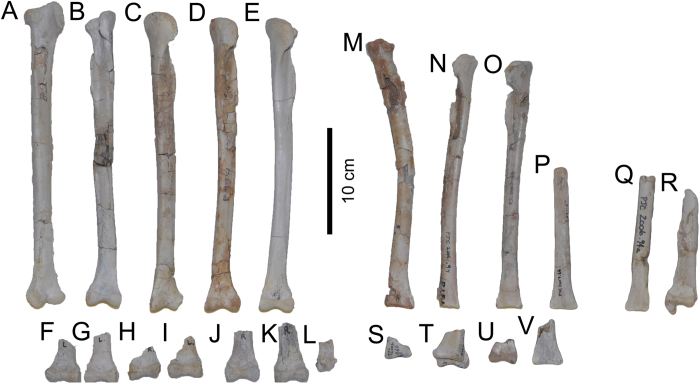
*Avimimus*. Tibiotarsi recovered from the *Avimimus* bonebed arranged by total length, demonstrating size dichotomy between tibiotarsi fused with astragalocalcanei (**A**–**L**) and tibiotarsi unfused to astragalocalcanei (**M**–**W**). MPC-D 102/92 (**A**); MPC-D 102/94 (**B**); MPC-D 102/42 (**C**); MPC-D 102/84 (**D**); MPC-D 102/90 (**E**); MPC-D 102/83 (**M**); MPC-D 102/74 (**N**); MPC-D 102/105 (**O**); MPC-D 102.86 (**P**); MPC-D 102/74a (**Q**); and MPC-D 102/26 (**R**) in anterior view.

**Figure 8:**
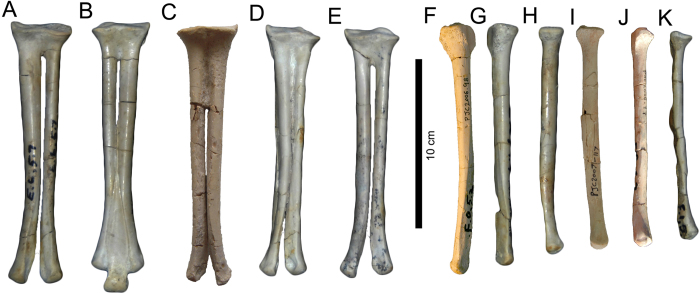
*Avimimus*. Metatarsi recovered from the *Avimimus* bonebed arranged by size, demonstrating size dichotomy between fused elements (**A–E**) and isolated elements (**F–K**). Note small degree of variation in length of fused tarsometatarsi. MPC-D 102/37 (**A**); MPC-D 102/89 (**B**); MPC-D 102/93 (**C**); MPC-D 102/76 (**D**); MPC-D 102/96 (**E**); MPC-D 102/78 (**F**); MPC-D 102/40 (**G**); MPC-D 102/48 (**H**); MPC-D 102/106 (**I**); MPC-D 102/77 (**J**); MPC-D 102/39 (**K**).

The predominance of thick-walled, hydrodynamically dense elements (Figs 7 and 8, Table S1) and the taphonomic signatures of the bones, combined with sedimentological and paleocurrent data, suggest that this assemblage represents a secondary deposit of previously buried skeletal material. The original death assemblage was probably formed by a catastrophic mass death event and the remains were then accumulated in the paleochannel during a second depositional event. The cause of the mass death cannot be determined with certainty, although the assemblage is somewhat similar to the ornithischian bonebeds from the Late Cretaceous of the Western Interior of North America. These include well-studied bonebeds such as the *Centrosaurus* bonebeds of Dinosaur Provincial Park^8^ and the *Pachyrhinosaurus* bonebeds of the Wapiti Formation^16^. These assemblages consist primarily of single species, although there are often isolated elements from other taxa. Larger individuals dominate, and assemblages are preserved as disarticulated, hydraulically sorted elements in channel lag deposits. These bonebeds are thought to result from the catastrophic deaths of many individuals in groups or herds, drowned during flooding events^8^,^11^,^16^. The high proportion of distal hindlimb elements is unusual, especially considering that other dense elements, like sacra and the fused pelvic elements^27^, are underrepresented (Fig. S1). This may point to a miring event as the cause of the mass death. Unfortunately, any sedimentological indications of miring have been erased by the second flow event, and therefore the cause of death is ambiguous.

The composition of the bonebed and the high number of individuals have implications for the behaviour of *Avimimus*. The death assemblage strongly suggests that *Avimimus* engaged in gregarious behaviour, although the particular nature of that behaviour is not clear. The morphology of the mandible (Fig. 6) in *Avimimus* is similar to oviraptorids and caenagnathids, which were probably herbivorous^44^,^45^, so it is unlikely that the bonebed is evidence of pack hunting or a scavenging-driven assemblage. The presence of more than two adults suggests that the bonebed is not an isolated family group, and the mix of subadults and adults in such a large aggregation argues against an assemblage of parents and their offspring. Other assemblages of multiple omnivorous or herbivorous theropods have been discovered, most notably therizinosaurs^46^ and ornithomimids^17^. Kirkland *et al*.^46^ studied a paucispecific bonebed of *Falcarius*, with more than 300 individuals and a range of developmental stages^47^. The abundance of material (>2000 specimens)^47^, and the 99% dominance of *Falcarius* at the site^46^ strongly suggest that the site is the result of a catastrophic mass death. The presence of multiple developmental stages indicates that the *Falcarius* assemblage reflects typical population structure, which suggests that it may represent a non-transient herd. This appears to be untrue of the *Avimimus* bonebed, which, despite a smaller sample, shows a strong bias in the presence of certain developmental stages. It is possible that the near absence of juveniles in the bonebed is the result of reproductive seasonality and rapid growth. In this case, young *Avimimus* are born at the same time of year and grow to near-adult size within a year. The result is a mixed-age assemblage of similar size. In the absence of histological data, however, this assertion cannot be tested, but the presence of one possibly juvenile individual (MPC-D 102/38) argues against it.

Although speculative, the paucity of juveniles in the bonebed may instead indicate that *Avimimus* formed age-segregated assemblages. These groups may have enjoyed reproductive, antipredator, or foraging benefits, but the contribution of these factors to the formation of the assemblages is unclear. Lekking behaviour, where individuals group to display to potential mates, is known in multiple groups^48^,^49^, especially birds. Aggregations may include as many as 100 individuals^50^ of varying age, size, and sexual fitness. The near absence of juveniles is consistent with, but not indicative of, a lekking assemblage. The sex of any individual in the assemblage cannot be assessed, and therefore the ratio of genders cannot be used to evaluate the hypothesis of lekking. Alternatively, the bonebed assemblage may be evidence of flocking or communal roosting behaviour for any number of reasons. The anti-predator effectiveness of flocking and communal roosting^51^ is documented especially well in birds^52^,^53^,^54^ and mammals^55^,^56^,^57^, among other animals. Multiple studies show that vigilance^58^ is reduced in larger groups, increasing foraging efficiency^59^,^60^. Kobayashi and Lu^17^ described an assemblage of at least 14 articulated *Sinornithomimus* from China, with a high proportion of juveniles^37^, which they suggested is the result of a predator avoidance strategy. However, in the absence of a larger sample size and evidence of the cause of the mass death event, the specific behaviour that the *Avimimus* death assemblage represents cannot be assessed.

Monodominant and monotaxic associations of bones and skeletons are known for many taxa of dinosaurs (Table S3). The distribution of these bonebeds is demonstrably non-random with respect to phylogeny and stratigraphy, although the effects of outcrop availability and sampling are unclear. Certain animals, especially during the Cretaceous period, show a strong tendency to form monodominant or monotaxic assemblages^11^. These include the hadrosaurine hadrosaurs, especially *Edmontosaurus*^9^, and centrosaurine ceratopsids^8^,^15^. Oviraptorosaurs can be added to this list, as numerous associations of multiple oviraptorosaurs are known, including *Anzu wyliei*^61^*, Conchoraptor gracilis* (MPC-D 102/3; M. A. Norell pers. comm.), *Heyuannia huangi*^62^, and *Khaan mckennai*^63^. Although it could be argued that this is simply an artifact resulting from the association between certain lineages and environments that are subject to flooding events, such as coastal plains, the fact remains that many dinosaurs and other taxa that inhabit these environments do not occur in such assemblages. Furthermore, many of the associations, especially those of oviraptorosaurs (*Conchoraptor, Khaan*), are not from fluvial sediments. In any case, the *Avimimus* bonebed provides the first strong evidence of gregarious behaviour in oviraptorosaurs, and improves our knowledge of social behaviour in maniraptorans.

## Methods

### Collection

The bonebed was excavated systematically and mapped (Fig. 3) using a 1 m^2^ grid system. Once mapped, specimens were collected and given coordinate numbers referring to their location within the map grid (see supplementary information). Plan view, bi-directional orientations (azimuthal trends) of elements were obtained from the map using ImageJ 1.48 v. These data were then plotted on a rose diagram divided into 45-degree quadrants (Fig. 3). Rao’s Spacing and Raleigh Z tests from the R software package “circular” were used to assess whether the *in situ* assemblage exhibited statistically significant preferred orientations. Specimens were mechanically prepared at the MPC by “Dinosaurs of the Gobi” participants, using a combination of manual and air-pressured tools and a variety of consolidants (Butvar, cyanoacrylate).

### Taphonomic analysis

Taphonomic bone-modification data^64^ were assessed through simple visual inspection of prepared elements. The adult skeleton of *Avimimus* is characterized by the fusion of many bones into compound elements, reducing the number of discrete skeletal elements through ontogeny. As such, the mixture of adults and subadults in this bonebed makes it difficult to accurately assess skeletal element representation. Skeletal representation was therefore calculated separately for both an adult skeleton and a subadult skeleton with no fused elements (Table S2). This provides minimum and maximum boundaries, although the true pattern is likely towards the maximum boundary, because compound elements were more commonly fused than unfused in the bonebed. Minimum number of individuals was estimated using the maximum number of unique elements (distal right tibiae). Combining this MNI (n = 13) with length estimates of partial tibiae based on the proportions of complete tibiae gave a better estimate of the number of tibiae of substantially different lengths (n = 18).

### Measurement

Calipers were used to measure small and medium size elements (<150 mm in maximum dimension) to an accuracy of 0.5 mm. For large elements measuring more than 150 mm in maximum dimension, a fabric measuring tape was employed. For MNI information, complete tibiae were measured and ratios of anteroposterior proximal width, transverse distal width, and transverse shaft diameter were correlated with tibia length. This allowed the lengths of partial tibiae to be estimated from other available measurements based on the proportions of complete tibiae. None of the length estimates from opposite sides were close in value, so it is unlikely that two tibiae from which lengths were estimated belonged to the same individual.

## Additional Information

**How to cite this article**: Funston, G. F. *et al*. The first oviraptorosaur (Dinosauria: Theropoda) bonebed: evidence of gregarious behaviour in a maniraptoran theropod. *Sci. Rep.* **6**, 35782; doi: 10.1038/srep35782 (2016).

## Supplementary Material

Supplementary Information

## Figures and Tables

**Table 1:** Selected measurements of tibiotarsi recovered from the *Avimimus* bonebed in the Nemegt Formation of Mongolia.

Specimen	Side	Fusion	T. length	T-ast L	T. pw	T. sw transverse	T. dw
MPC-D 102/69	Left	No	228e				26.8
MPC-D 102/105	Right	No	230		36.5	13.6	27
MPC-D 102/67	Right	No	231e				27.9
MPC-D 102/74	Right	No	232		34.9	16	23+
MPC-D 102/74A	Left	No	232e			14.4	
MPC-D 102/26	Right	No	236e			15.7	26.8
MPC-D 102/24	Left	No	238e				28.7
MPC-D 102/47	Right	Suture	239e				27
MPC-D 102/68	Left	No	240e				28.3
MPC-D 102/51	Right	No	244e			15.6	29e
MPC-D 102/83	Right	No	242		36	14.4	31.1
MPC-D 102/52	Right	Yes	246e				27.7
MPC-D 102/90	Left	Yes	259	264		15.3	30.5
MPC-D 102/84	Left	Yes	260	262	42.5	16.7	30.1
MPC-D 102/42	Left	Yes	264	269	43.1	16.1	31.7
MPC-D 102/94	Right	Yes	260	262	40		27.9
MPC-D 102/23	Left	Yes	265e				33.8
MPC-D 102/25	Left	Yes	265e				30
MPC-D 102/19	Right	Yes	266e				30.3
MPC-D 102/22	Left	Yes	266e				31.4
MPC-D 102/92	Right	Yes	278	282	46		30
MPC-D 102/18	Right	Yes	280e				31.7
MPC-D 102/17	Right	Yes	280e				31.2
MPC-D 102/102	Right	Yes	280e				17+
MPC-D 102/15	Right	?	254e		40.8		
MPC-D 102/27	Left	?	232e		32+		
MPC-D 102/62	Right	?	254e		40.6		
MPC-D 102/63	Right	?	245e		38.1		
MPC-D 102/66	Right	?	242e		37.4		
MPC-D 102/72	Right	?	273e		46.3		
MPC-D 102/38	Left	?	202e		26.9		
MPC-D 102/44	Left	?	235e		35.4		
MPC-D 102/53	Left	?	267e		44.5		

^*^e: estimate,

^+^Measurement likely greater than indicated.
