# Supplementary material for: The first oviraptorosaur (Dinosauria: Theropoda) bonebed: evidence of gregarious behaviour in a maniraptoran theropod
Source: Sci Rep. 2016 Oct 21;6:35782. doi: 10.1038/srep35782 (PMC5073311; doi:10.1038/srep35782)
Supplement: Supplementary Information [file srep35782-s1.pdf]

Supplementary Information

The first oviraptorosaur (Dinosauria: Theropoda) bonebed: evidence of gregarious behaviour in a maniraptoran theropod

Gregory F. Funston[1]*, Philip J. Currie[1], David A. Eberth[2], Michael J. Ryan[3], Tsogtbaatar Chinzorig[4], Demchig Badamgarav[5]†, Nicholas R. Longrich[6],

[1] University of Alberta, Department of Biological Sciences, CW405 Biological Sciences Building, Edmonton, Alberta, T6G 2E9, Canada, (780) 492-1252; (780) 492-9234 funston@ualberta.ca
[2] Royal Tyrrell Museum of Palaeontology, Box 7500, Drumheller, Alberta, T0J 0Y0 Canada
[3] Department of Vertebrate Paleontology, Cleveland Museum of Natural History, 1 Wade Oval Dr., University Circle, Cleveland, OH 44106, USA
[4] Hokkaido University Museum, Hokkaido University, Sapporo 060-0810, Japan
[5] Paleontological Center, Mongolian Academy of Sciences, Box 260, Ulaan Baatar 210351, Mongolia
[6] Department of Biology and Biochemistry, University of Bath, Claverton Down, Bath, United Kingdom

*Corresponding Author
† Deceased

Table S1: Catalog of *Avimimus* specimens from the *Avimimus* bonebed in the Nemegt Formation of Mongolia.

MPC number	Specimen #	Date Found	Taxon	Element	Found by	Locality	Formation	North	East
**MPC-D 102/15**	PJC2006.001	Aug. 17, 2006	*Avimimus*	right tibia proximal end and shaft	Nick Longrich	Nemegt	Nemegt	43deg 28.891min	101deg 4.093min
MPC-D 102/101	PJC2006.002	Aug. 17, 2006	*Avimimus*	cervical vertebra	Nick Longrich	Nemegt	Nemegt	43deg 28.891min	101deg 4.093min
**MPC-D 102/16**	PJC2006.003	Aug. 17, 2006	*Avimimus*	dentaries	Nick Longrich	Nemegt	Nemegt	43deg 28.891min	101deg 4.093min
	PJC2006.004	Aug. 17, 2006	*Avimimus*	pedal phalanx	Nick Longrich	Nemegt	Nemegt	43deg 28.891min	101deg 4.093min
	PJC2006.005	Aug. 17, 2006	*Avimimus*	pedal phalanx	Nick Longrich	Nemegt	Nemegt	43deg 28.891min	101deg 4.093min
**MPC-D 102/17**	PJC2006.006	Aug. 17, 2006	*Avimimus*	right astragalus and tibia distal end	Nick Longrich	Nemegt	Nemegt	43deg 28.891min	101deg 4.093min
**MPC-D 102/18**	PJC2006.007	Aug. 17, 2006	*Avimimus*	right astragalus and tibia distal end	Nick Longrich	Nemegt	Nemegt	43deg 28.891min	101deg 4.093min
**MPC-D 102/19**	PJC2006.008	Aug. 17, 2006	*Avimimus*	right astragalus and tibia distal end	Nick Longrich	Nemegt	Nemegt	43deg 28.891min	101deg 4.093min
**MPC-D 102/20**	PJC2006.009	Aug. 17, 2006	*Avimimus*	right astragalus and tibia distal end	Nick Longrich	Nemegt	Nemegt	43deg 28.891min	101deg 4.093min
**MPC-D 102/21**	PJC2006.010	Aug. 17, 2006	*Avimimus*	metatarsals shafts II & IV	Nick Longrich	Nemegt	Nemegt	43deg 28.891min	101deg 4.093min
	PJC2006.011	Aug. 17, 2006	*Avimimus*	phalanx	Nick Longrich	Nemegt	Nemegt	43deg 28.891min	101deg 4.093min
**MPC-D 102/22**	PJC2006.012	Aug. 17, 2006	*Avimimus*	left astragalus and distal tibia	Nick Longrich	Nemegt	Nemegt	43deg 28.891min	101deg 4.093min
**MPC-D 102/23**	PJC2006.013	Aug. 17, 2006	*Avimimus*	left astragalus and distal tibia	Nick Longrich	Nemegt	Nemegt	43deg 28.891min	101deg 4.093min
**MPC-D 102/24**	PJC2006.014	Aug. 17, 2006	*Avimimus*	left astragalus and distal tibia	Nick Longrich	Nemegt	Nemegt	43deg 28.891min	101deg 4.093min
**MPC-D 102/25**	PJC2006.015	Aug. 17, 2006	*Avimimus*	left astragalus and distal tibia	Nick Longrich	Nemegt	Nemegt	43deg 28.891min	101deg 4.093min
**MPC-D 102/26**	PJC2006.016	Aug. 17, 2006	*Avimimus*	right tibia, fibula, astragalus, calcaneum	Nick Longrich	Nemegt	Nemegt	43deg 28.891min	101deg 4.093min
	PJC2006.017	Aug. 17, 2006	*Avimimus*	vertebra	Nick Longrich	Nemegt	Nemegt	43deg 28.891min	101deg 4.093min
	PJC2006.018	Aug. 17, 2006	*Avimimus*	phalanx plus ?	Nick Longrich	Nemegt	Nemegt	43deg 28.891min	101deg 4.093min
	PJC2006.022	Aug. 18, 2006	*Avimimus*	rib head	Crew	Nemegt	Nemegt	43deg 28.891min	101deg 4.093min
	PJC2006.023	Aug. 18, 2006	*Avimimus*	rib	Crew	Nemegt	Nemegt	43deg 28.891min	101deg 4.093min
**MPC-D 102/27**	PJC2006.024	Aug. 18, 2006	*Avimimus*	femur left distal end tibia left prox. end	Nick Longrich	Nemegt	Nemegt	43deg 28.891min	101deg 4.093min

**MPC-D 102/28**	PJC2006.025	Aug. 18, 2006	*Avimimus*	right proximal tarsometatarsus	Crew	Nemegt	Nemegt	43deg 28.891min	101deg 4.093min
**MPC-D 102/29**	PJC2006.026	Aug. 18, 2006	*Avimimus*	left proximal tarsometatarsus	Crew	Nemegt	Nemegt	43deg 28.891min	101deg 4.093min
**MPC-D 102/30**	PJC2006.027	Aug. 18, 2006	*Avimimus*	right proximal tarsometatarsus	Crew	Nemegt	Nemegt	43deg 28.891min	101deg 4.093min
**MPC-D 102/31**	PJC2006.028	Aug. 18, 2006	*Avimimus*	left proximal tarsometatarsus	Crew	Nemegt	Nemegt	43deg 28.891min	101deg 4.093min
**MPC-D 102/32**	PJC2006.029	Aug. 18, 2006	*Avimimus*	right proximal tarsometatarsus	Crew	Nemegt	Nemegt	43deg 28.891min	101deg 4.093min
**MPC-D 102/33**	PJC2006.029	Aug. 19, 2006	*Avimimus*	skull frag ?	Eva B. Koppelhus	Nemegt	Nemegt	43deg 28.891min	101deg 4.093min
MPC-D 102/102	PJC2006.044	Aug. 19, 2006	*Avimimus*	cervical vertebra					
	PJC2006.050	Aug. 18, 2006	*Avimimus*	phalanx	Crew	Nemegt	Nemegt	43deg 28.891min	101deg 4.093min
	PJC2006.051	Aug. 18, 2006	*Avimimus*	fibula shaft	Crew	Nemegt	Nemegt	43deg 28.891min	101deg 4.093min
**MPC-D 102/34**	PJC2006.059	Aug. 19, 2006		partial skull					
**MPC-D 102/35**	PJC2006.104	Aug. 19, 2006	*Avimimus*	metatarsal II	Crew	Nemegt	Nemegt	43deg 28.891min	101deg 4.093min
**MPC-D 102/36**	PJC2006.105	Aug. 20, 2006	*Avimimus*	femur, left complete	Crew	Nemegt	Nemegt	43deg 28.891min	101deg 4.093min
	PJC2006.106	Aug. 20, 2006	*Avimimus*	ungual	Crew	Nemegt	Nemegt	43deg 28.891min	101deg 4.093min
	PJC2006.107	Aug. 20, 2006	*Avimimus*	rib	Crew	Nemegt	Nemegt	43deg 28.891min	101deg 4.093min
	PJC2006.108	Aug. 20, 2006	*Avimimus*	distal end of metatarsal	Crew	Nemegt	Nemegt	43deg 28.891min	101deg 4.093min
	PJC2006.109	Aug. 20, 2006	*Avimimus*	pedal phalanx	Crew	Nemegt	Nemegt	43deg 28.891min	101deg 4.093min
	PJC2006.110	Aug. 20, 2006	*Avimimus*	pedal phalanx	Crew	Nemegt	Nemegt	43deg 28.891min	101deg 4.093min
	PJC2006.111	Aug. 20, 2006	*Avimimus*	phalanx distal end	Crew	Nemegt	Nemegt	43deg 28.891min	101deg 4.093min
	PJC2006.112	Aug. 20, 2006	*Avimimus*	vertebra	Crew	Nemegt	Nemegt	43deg 28.891min	101deg 4.093min
**MPC-D 102/37**	PJC2006.113	Aug. 20, 2006	*Avimimus*	metatarsal right	Crew	Nemegt	Nemegt	43deg 28.891min	101deg 4.093min
**MPC-D 102/38**	PJC2006.114	Aug. 20, 2006	*Avimimus*	left tibia incomplete	Crew	Nemegt	Nemegt	43deg 28.891min	101deg 4.093min
**MPC-D 102/39**	PJC2006.115	Aug. 20, 2006	*Avimimus*	metatarsal II right	Crew	Nemegt	Nemegt	43deg 28.891min	101deg 4.093min
**MPC-D**	PJC2006.116	Aug. 20,	*Avimimus*	metatarsal IV left	Crew	Nemegt	Nemegt	43deg 28.891min	101deg 4.093min

**102/40**		2006							
**MPC-D 102/41**	PJC2006.117	Aug. 20, 2006	*Avimimus*	metatarsal IV right	Crew	Nemegt	Nemegt	43deg 28.891min	101deg 4.093min
**MPC-D 102/42**	PJC2006.118	Aug. 20, 2006	*Avimimus*	tibia + fragments in a small plastic bag	Crew	Nemegt	Nemegt	43deg 28.891min	101deg 4.093min
	PJC2006.119	Aug. 20, 2006	*Avimimus*	fibula	Crew	Nemegt	Nemegt	43deg 28.891min	101deg 4.093min
	PJC2006.120	Aug. 20, 2006	*Avimimus*	phalanx	Crew	Nemegt	Nemegt	43deg 28.891min	101deg 4.093min
**MPC-D 102/43**	PJC2006.122	Aug. 20, 2006	*Avimimus*	metatarsal	Crew	Nemegt	Nemegt	43deg 28.891min	101deg 4.093min
	PJC2006.123	Aug. 20,2006	*Avimimus*	rib	Crew	Nemegt	Nemegt	43deg 28.891min	101deg 4.093min
	PJC2006.124	Aug. 20, 2006	*Avimimus*	rib head	Crew	Nemegt	Nemegt	43deg 28.891min	101deg 4.093min
**MPC-D 102/44**	PJC2006.125	Aug. 20, 2006	*Avimimus*	tibia head	Crew	Nemegt	Nemegt	43deg 28.891min	101deg 4.093min
**MPC-D 102/45**	PJC2006.126	Aug. 20, 2006	*Avimimus*	metarsal III	Crew	Nemegt	Nemegt	43deg 28.891min	101deg 4.093min
**MPC-D 102/46**	PJC2006.127	Aug. 20, 2006	*Avimimus*	tarsometatarsus proximal end left	Crew	Nemegt	Nemegt	43deg 28.891min	101deg 4.093min
**MPC-D 102/47**	PJC2006.128	Aug. 20, 2006	*Avimimus*	distal end of tibia + astragalus right	Crew	Nemegt	Nemegt	43deg 28.891min	101deg 4.093min
**MPC-D 102/48**	PJC2006.129	Aug. 20, 2006	*Avimimus*	metatarsal	Crew	Nemegt	Nemegt	43deg 28.891min	101deg 4.093min
	PJC2006.130	Aug. 20, 2006	*Avimimus*	ischium	Crew	Nemegt	Nemegt	43deg 28.891min	101deg 4.093min
	PJC2006.131	Aug. 20, 2006	*Avimimus*	ilium in three pieces	Crew	Nemegt	Nemegt	43deg 28.891min	101deg 4.093min
MPC-D 102/103	PJC2006.164	Aug. 20, 2006	*Avimimus*	cervical vertebra	Crew	Nemegt	Nemegt	43deg 28.891min	101deg 4.093min
**MPC-D 102/49**	PJC2006.165	Aug. 20, 2006	*Avimimus*	pubis	Crew	Nemegt	Nemegt	43deg 28.891min	101deg 4.093min
	PJC2006.166	Aug. 20, 2006	*Avimimus*	fibula	Crew	Nemegt	Nemegt	43deg 28.891min	101deg 4.093min
	PJC2006.167	Aug. 20, 2006	*Avimimus*	fibula	Crew	Nemegt	Nemegt	43deg 28.891min	101deg 4.093min
MPC-D 102/104	PJC2006.168	Aug. 20, 2006	*Avimimus*	cervical neural arch	Crew	Nemegt	Nemegt	43deg 28.891min	101deg 4.093min
	PJC2006.169	Aug. 20, 2006	*Avimimus*	forelimb	Crew	Nemegt	Nemegt	43deg 28.891min	101deg 4.093min
**MPC-D 102/50**	PJC2006.170	Aug. 20, 2006	*Avimimus*	pubic boot	Crew	Nemegt	Nemegt	43deg 28.891min	101deg 4.093min
**MPC-D 102/51**	PJC2006.171	Aug. 20, 2006	*Avimimus*	right tibia (partial)	Crew	Nemegt	Nemegt	43deg 28.891min	101deg 4.093min

**MPC-D 102/52**	PJC2006.172	Aug. 20, 2006	*Avimimus*	distal end of right tibia	Crew	Nemegt	Nemegt	43deg 28.891min	101deg 4.093min
**MPC-D 102/53**	PJC2006.176	Aug. 22, 2006	*Avimimus*	proximal end of tibia	Crew	Nemegt	Nemegt	43deg 28.891min	101deg 4.093min
**MPC-D 102/54**	PJC2006.177	Aug. 22, 2006	*Avimimus*	tarsometatarsal only proximal end	Crew	Nemegt	Nemegt	43deg 28.891min	101deg 4.093min
**MPC-D 102/55**	PJC2006.178	Aug. 22, 2006	*Avimimus*	proximal end of humerus	Crew	Nemegt	Nemegt	43deg 28.891min	101deg 4.093min
**MPC-D 102/56**	PJC2006.30	Aug. 18, 2006	*Avimimus*	right proximal tarsometatarsus	Crew	Nemegt	Nemegt	43deg 28.891min	101deg 4.093min
**MPC-D 102/57**	PJC2006.31	Aug. 18, 2006	*Avimimus*	right proximal tarsometatarsus ???	Crew	Nemegt	Nemegt	43deg 28.891min	101deg 4.093min
**MPC-D 102/58**	PJC2006.32	Aug. 18, 2006	*Avimimus*	right proximal tarsometatarsus fragment	Crew	Nemegt	Nemegt	43deg 28.891min	101deg 4.093min
**MPC-D 102/59**	PJC2006.33	Aug. 18, 2006	*Avimimus*	right proximal tarsometatarsus	Michael Ryan	Nemegt	Nemegt	43deg 28.891min	101deg 4.093min
**MPC-D 102/60**	PJC2006.34	Aug. 18, 2006	*Avimimus*	shaft of mt III	Crew	Nemegt	Nemegt	43deg 28.891min	101deg 4.093min
**MPC-D 102/61**	PJC2006.35	Aug. 18, 2006	*Avimimus*	distal end of mt III	Crew	Nemegt	Nemegt	43deg 28.891min	101deg 4.093min
**MPC-D 102/62**	PJC2006.36	Aug. 18, 2006	*Avimimus*	right tibia proximal end	Crew	Nemegt	Nemegt	43deg 28.891min	101deg 4.093min
**MPC-D 102/63**	PJC2006.37	Aug. 18, 2006	*Avimimus*	right tibia proximal end	Crew	Nemegt	Nemegt	43deg 28.891min	101deg 4.093min
	PJC2006.38	Aug. 18, 2006	*Avimimus*	right femur distal + ?	Crew	Nemegt	Nemegt	43deg 28.891min	101deg 4.093min
	PJC2006.39	Aug. 18, 2006	*Avimimus*	right femur proximal end	Crew	Nemegt	Nemegt	43deg 28.891min	101deg 4.093min
	PJC2006.40	Aug. 18, 2006	*Avimimus*	right femur distal end	Crew	Nemegt	Nemegt	43deg 28.891min	101deg 4.093min
	PJC2006.41	Aug. 18, 2006	*Avimimus*	left femur distal end	Crew	Nemegt	Nemegt	43deg 28.891min	101deg 4.093min
	PJC2006.42	Aug. 18, 2006	*Avimimus*	pedal phalanx IV-I	Crew	Nemegt	Nemegt	43deg 28.891min	101deg 4.093min
	PJC2006.43	Aug. 18, 2006	*Avimimus*	pedal phalanx	Crew	Nemegt	Nemegt	43deg 28.891min	101deg 4.093min
	PJC2006.44	Aug. 18, 2006	*Avimimus*	cervical vertebra	Crew	Nemegt	Nemegt	43deg 28.891min	101deg 4.093min
	PJC2006.45	Aug. 18, 2006	*Avimimus*	vertebra	Crew	Nemegt	Nemegt	43deg 28.891min	101deg 4.093min
**MPC-D 102/64**	PJC2006.46	Aug. 18, 2006	*Avimimus*	nasals	Crew	Nemegt	Nemegt	43deg 28.891min	101deg 4.093min
**MPC-D 102/65**	PJC2006.47	Aug. 18, 2006	*Avimimus*	distal end right? Astragalus + tibia	Crew	Nemegt	Nemegt	43deg 28.891min	101deg 4.093min
	PJC2006.48	Aug. 18,	*Avimimus*	phalanx	Crew	Nemegt	Nemegt	43deg 28.891min	101deg 4.093min

		2006							
**MPC-D 102/66**	PJC2006.49	Aug. 18, 2006	*Avimimus*	proximal end of right tibia	Crew	Nemegt	Nemegt	43deg 28.891min	101deg 4.093min
**MPC-D 102/67**	PJC2006.60	Aug. 19, 2006	*Avimimus*	distal end of tibia	Crew	Nemegt	Nemegt	43deg 28.891min	101deg 4.093min
	PJC2006.61	Aug. 19, 2006	*Avimimus*	partial heads of limbbones	Crew	Nemegt	Nemegt	43deg 28.891min	101deg 4.093min
	PJC2006.62	Aug. 19, 2006	*Avimimus*	pedal phalanx II-I	Crew	Nemegt	Nemegt	43deg 28.891min	101deg 4.093min
	PJC2006.63	Aug. 19, 2006	*Avimimus*	distal end of metatarsal ? II	Crew	Nemegt	Nemegt	43deg 28.891min	101deg 4.093min
	PJC2006.64	Aug. 19, 2006	*Avimimus*	? Pedal phalanx	Crew	Nemegt	Nemegt	43deg 28.891min	101deg 4.093min
**MPC-D 102/68**	PJC2006.65	Aug. 19, 2006	*Avimimus*	distal end of right tibia	Crew	Nemegt	Nemegt	43deg 28.891min	101deg 4.093min
**MPC-D 102/69**	PJC2006.66	Aug. 19, 2006	*Avimimus*	pedal phalanx IV-4	Crew	Nemegt	Nemegt	43deg 28.891min	101deg 4.093min
	PJC2006.67	Aug. 19, 2006	*Avimimus*	pedal phalanx	Crew	Nemegt	Nemegt	43deg 28.891min	101deg 4.093min
	PJC2006.68	Aug. 19, 2006	*Avimimus*	pedal phalanx III-3?	Crew	Nemegt	Nemegt	43deg 28.891min	101deg 4.093min
	PJC2006.69	Aug. 19, 2006	*Avimimus*	left femur proximal end	Crew	Nemegt	Nemegt	43deg 28.891min	101deg 4.093min
	PJC2006.70	Aug. 19, 2006	*Avimimus*	manual phalanx	Crew	Nemegt	Nemegt	43deg 28.891min	101deg 4.093min
**MPC-D 102/70**	PJC2006.71	Aug. 19, 2006	*Avimimus*	distal end of both pubes	Crew	Nemegt	Nemegt	43deg 28.891min	101deg 4.093min
	PJC2006.72	Aug. 19, 2006	*Avimimus*	proximal end of metatarsal II	Crew	Nemegt	Nemegt	43deg 28.891min	101deg 4.093min
	PJC2006.73	Aug. 19, 2006	*Avimimus*	sacral centrum	Crew	Nemegt	Nemegt	43deg 28.891min	101deg 4.093min
	PJC2006.74	Aug. 19, 2006	*Avimimus*	distal end of metatarsal ? II	Crew	Nemegt	Nemegt	43deg 28.891min	101deg 4.093min
	PJC2006.75	Aug. 19, 2006	*Avimimus*	distal end metatarsal III	Crew	Nemegt	Nemegt	43deg 28.891min	101deg 4.093min
	PJC2006.76	Aug. 19, 2006	*Avimimus*	manual phalanx	Crew	Nemegt	Nemegt	43deg 28.891min	101deg 4.093min
	PJC2006.77	Aug. 19, 2006	*Avimimus*	distal end of metatarsal IV two of them	Crew	Nemegt	Nemegt	43deg 28.891min	101deg 4.093min
	PJC2006.78	Aug. 19, 2006	*Avimimus*	pedal phalanx proximal	Crew	Nemegt	Nemegt	43deg 28.891min	101deg 4.093min
	PJC2006.79	Aug. 19, 2006	*Avimimus*	phalanx	Crew	Nemegt	Nemegt	43deg 28.891min	101deg 4.093min
	PJC2006.80	Aug. 19, 2006	*Avimimus*	pedal ungual III-4	Crew	Nemegt	Nemegt	43deg 28.891min	101deg 4.093min

	PJC2006.81	Aug. 19, 2006	*Avimimus*	?radius	Crew	Nemegt	Nemegt	43deg 28.891min	101deg 4.093min
	PJC2006.82	Aug. 19, 2006	*Avimimus*	pedal phalanx	Crew	Nemegt	Nemegt	43deg 28.891min	101deg 4.093min
	PJC2006.83	Aug. 19, 2006	*Avimimus*	femur, left distal end	Crew	Nemegt	Nemegt	43deg 28.891min	101deg 4.093min
**MPC-D 102/71**	PJC2006.84	Aug. 19, 2006	*Avimimus*	metatarsal III	Crew	Nemegt	Nemegt	43deg 28.891min	101deg 4.093min
	PJC2006.85	Aug. 19, 2006	*Avimimus*	head of femur	Crew	Nemegt	Nemegt	43deg 28.891min	101deg 4.093min
	PJC2006.86	Aug. 19, 2006	*Avimimus*	proximal tibia right	Crew	Nemegt	Nemegt	43deg 28.891min	101deg 4.093min
**MPC-D 102/72**	PJC2006.87	Aug. 19, 2006	*Avimimus*	proximal tibia right	Crew	Nemegt	Nemegt	43deg 28.891min	101deg 4.093min
**MPC-D 102/73**	PJC2006.88	Aug. 19, 2006	*Avimimus*	pubis shaft	Crew	Nemegt	Nemegt	43deg 28.891min	101deg 4.093min
	PJC2006.89	Aug. 19, 2006	*Avimimus*	? Radius	Crew	Nemegt	Nemegt	43deg 28.891min	101deg 4.093min
	PJC2006.90	Aug. 19, 2006	*Avimimus*	pedal ungual	Crew	Nemegt	Nemegt	43deg 28.891min	101deg 4.093min
	PJC2006.91	Aug. 19, 2006	*Avimimus*	pedal phalanx	Crew	Nemegt	Nemegt	43deg 28.891min	101deg 4.093min
	PJC2006.92	Aug. 19, 2006	*Avimimus*	tarsal	Crew	Nemegt	Nemegt	43deg 28.891min	101deg 4.093min
	PJC2006.93	Aug. 19, 2006	*Avimimus*	rib	Crew	Nemegt	Nemegt	43deg 28.891min	101deg 4.093min
**MPC-D 102/74**	PJC2006.94	Aug. 19, 2006	*Avimimus*	tibia + fragments in a small plastic bag	Crew	Nemegt	Nemegt	43deg 28.891min	101deg 4.093min
**MPC-D 102/75**	PJC2006.95	Aug. 19, 2006	*Avimimus*	proximal end of metatarsal	Crew	Nemegt	Nemegt	43deg 28.891min	101deg 4.093min
**MPC-D 102/76**	PJC2006.96	Aug. 19, 2006	*Avimimus*	metatarsals II & IV	Crew	Nemegt	Nemegt	43deg 28.891min	101deg 4.093min
**MPC-D 102/77**	PJC2006.97	Aug. 19, 2006	*Avimimus*	metatarsal	Crew	Nemegt	Nemegt	43deg 28.891min	101deg 4.093min
**MPC-D 102/78**	PJC2006.98	Aug. 19, 2006	*Avimimus*	metatarsal	Crew	Nemegt	Nemegt	43deg 28.891min	101deg 4.093min
**MPC-D 102/79**	PJC2006.99	Aug. 19, 2006	*Avimimus*	distal end of metatarsal IV	Crew	Nemegt	Nemegt	43deg 28.891min	101deg 4.093min
**MPC-D 102/80**	PJC2007.002	2007-08-17	*avimimid*	fused metatarsals	Susan Kagan	West Sayr	Nemegt Fm	43.29.968	101.02.563
	PJC2007.023	2007-08-17	*avimimid*	pedal phalanx	Nick Longrich		Nemegt Fm	43.30.004	101.03.045
	PJC2007.023	2007-08-18	*avimimid*	miscellaneous elements	Nick Longrich	Eastern Sayr	Barun Goyot Fm	43.29.780	101.03.792
	PJC2007.100	2007-08-	*Avimimus*	fibula	Victoria Arbour	avimimid	Nemegt	43.28.891	101.04.093

		21				bonebed	Fm		
	PJC2007.101	2007-08-21	*Avimimus*	fibula	Victoria Arbour	avimimid bonebed	Nemegt Fm	43.28.891	101.04.093
**MPC-D 102/81**	PJC2007.102	2007-08-21	*Avimimus*	partial skull	Crew	avimimid bonebed	Nemegt fm		
	PJC2007.103	2007-08-21	*Avimimus*	pedal ungual	Michael Ryan	avimimid bonebed	Nemegt Fm	43.28.891	101.04.093
**MPC-D 102/82**	PJC2007.108	2007-08-19	*Avimimus*	third metatarsal	crew	avimimid bonebed	Nemegt Fm	43.28.891	101.04.093
	PJC2007.109	2007-08-19	*Avimimus*	vertebral fragments, not necessarily from same vertebra	crew	avimimid bonebed	Nemegt Fm	43.28.891	101.04.093
	PJC2007.111	2007-08-19	*Avimimus*	tibia	Ito Yoshio	Western Sayr	Nemegt Fm	no coordinates	no coordinates
**MPC-D 102/83**	PJC2007.112	2007-08-21	*Avimimus*	tibia (right)	Ito Yoshio	avimimid bonebed	Nemegt Fm	43.28.891	101.04.093
**MPC-D 102/84**	PJC2007.115	2007-08-21	*Avimimus*	tibia	crew	avimimid bonebed	Nemegt Fm	43.28.891	101.04.093
	PJC2007.116	2007-08-21	*Avimimus*	fibula	crew	avimimid bonebed	Nemegt Fm	43.28.891	101.04.093
**MPC-D 102/106**	PJC2007.117	2007-08-21	*Avimimus*	metatarsal	crew	avimimid bonebed	Nemegt Fm	43.28.891	101.04.093
	PJC2007.61	2007-08-19	*Avimimus*	phalanx	Astrid Schelde	avimimid bonebed	Nemegt Fm	43.28.891	101.04.093
	PJC2007.62	2007-08-19	*Avimimus*	rib	crew	avimimid bonebed	Nemegt Fm	43.28.891	101.04.093
	PJC2007.63	2007-08-19	*Avimimus*	centrum	Clive Coy	avimimid bonebed	Nemegt Fm	43.28.891	101.04.093
	PJC2007.64	2007-08-19	*Avimimus*	caudal vertebra	Clive Coy	avimimid bonebed	Nemegt Fm	43.28.891	101.04.093
**MPC-D 102/85**	PJC2007.65	2007-08-19	*Avimimus*	metatarsal	crew	avimimid bonebed	Nemegt Fm	43.28.891	101.04.093
**MPC-D 102/86**	PJC2007.66	2007-08-19	*Avimimus*	2 metatarsals	crew	avimimid bonebed	Nemegt Fm	43.28.891	101.04.093
**MPC-D 102/87**	PJC2007.67	2007-08-19	*Avimimus*	metatarsal	crew	avimimid bonebed	Nemegt Fm	43.28.891	101.04.093
	PJC2007.68	2007-08-19	*Avimimus*	limb element fragment	crew	avimimid bonebed	Nemegt Fm	43.28.891	101.04.093
**MPC-D 102/88**	PJC2007.69	2007-08-19	*Avimimus*	metatarsal	crew	avimimid bonebed	Nemegt Fm	43.28.891	101.04.093
**MPC-D 102/89**	PJC2007.70	2007-08-19	*Avimimus*	metatarsal	Julia Sankey	avimimid bonebed	Nemegt Fm	43.28.891	101.04.093
**MPC-D 102/90**	PJC2007.71	2007-08-19	*Avimimus*	tibia	crew	avimimid bonebed	Nemegt Fm	43.28.891	101.04.093
**MPC-D**	PJC2007.72	2007-08-	*Avimimus*	metatarsal III	crew	avimimid	Nemegt	43.28.891	101.04.093

**102/91**		19				bonebed	Fm		
**MPC-D 102/92**	PJC2007.73	2007-08-19	*Avimimus*	tibia plus fibula, astragalus, calcaneum	Astrid Schelde	avimimid bonebed	Nemegt Fm	43.28.891	101.04.093
**MPC-D 102/93**	PJC2007.74	2007-08-19	*Avimimus*	metatarsal	crew	avimimid bonebed	Nemegt Fm	43.28.891	101.04.093
**MPC-D 102/94**	PJC2007.86	2007-08-19	*Avimimus*	right tibia	Michael Ryan	avimimid bonebed	Nemegt Fm	43.28.891	101.04.093
**MPC-D 102/95**	PJC2007.87	2007-08-21	*Avimimus*	metatarsal	crew	avimimid bonebed	Nemegt Fm	43.28.891	101.04.093
**MPC-D 102/105**	PJC2007.88	2007-08-21	*Avimimus*	tibia	crew	avimimid bonebed	Nemegt Fm	43.28.891	101.04.093
**MPC-D 102/96**	PJC2007.89	2007-08-21	*Avimimus*	fused metatarsals 2 and 4	Astrid Schelde	avimimid bonebed	Nemegt Fm	43.28.891	101.04.093
	PJC2007.90	2007-08-21	*Avimimus*	fibula	Astrid Schelde	avimimid bonebed	Nemegt Fm	43.28.891	101.04.093
	PJC2007.92	2007-08-21	*Avimimus*	femur	Ito Yoshio	avimimid bonebed	Nemegt Fm	43.28.891	101.04.093
**MPC-D 102/97**	PJC2007.93	2007-08-21	*Avimimus*	metatarsal	Ito Yoshio	avimimid bonebed	Nemegt Fm	43.28.891	101.04.093
**MPC-D 102/98**	PJC2007.98	2007-08-21	*Avimimus*	metatarsal	Michael Ryan	avimimid bonebed	Nemegt Fm	43.28.891	101.04.093
**MPC-D 102/99**	PJC2007.99	2007-08-21	*Avimimus*	metatarsal	Ito Yoshio	avimimid bonebed	Nemegt Fm	43.28.891	101.04.093

Table S2: Skeletal representation at the *Avimimus* bonebed, calculated separately for an individual with unfused compound elements (above the break) and fused compound elements (below the break). Abbreviations: FE-TI-FIB: Femora, tibiae, fibulae; MTT-TARS: Metatarsals and tarsals; PELVIS-SACR: Pelvic elements and sacral vertebrae; HU-RAD-UL-CAR-MTC: forelimb elements including humeri, radii, ulnae, carpals and metacarpals; SCAP-COR: Pectoral girdle elements including scapulae, coracoids, sternals, and furculae.

Element category	**FE-TI-FIB**	**MTT-TARS**	**DENTARIES**	**PELVIS-SACR**	**PHALANGES**	**CRANIAL**	**VERTEBRAE**	**RIBS**	**HU-RAD-UL-CAR-MTC**	**SCAP-COR**		**TOTALS**
Collected	55	68	2	6	28	5	11	7	4	0		**186**
In one individual	*8*	*12*	*2*	*7*	*46*	*53*	*50*	*44*	*18*	*6*		**246**
Expected in an MNI of 18 individuals	144	216	36	126	828	954	900	792	324	108		**4428**
Skeletal Representation at AVBB (%)	**38%**	**31%**	**6%**	**5%**	**3%**	**1%**	**1%**	**1%**	**1%**	**0%**		**4%**
Over-, Average-, Under-representation*	**Over**	**Over**	**Over**	**Over**	**Under**	**Under**	**Under**	**Under**	**Under**	**Under**		
In one fused individual	6	4	2	3	46	32	50	44	8	3		**198**
Expected in an MNI of 18 individuals	108	72	36	54	828	576	900	792	144	54		**3564**
Skeletal Representation at AVBB (%)	**51%**	**94%**	**6%**	**11%**	**3%**	**1%**	**1%**	**1%**	**3%**	**0%**		**5%**
Over-, Average-, Under-representation*	**Over**	**Over**	**Over**	**Over**	**Under**	**Under**	**Under**	**Under**	**Under**	**Under**		

Table S3: List of known bonebed occurrences of dinosaur taxa by family.

Family	Taxon	Locality	Interpretation	Age	Reference
**Ceratopsidae**	*Centrosaurus apertus*	Dinosaur Provincial Park, AB	Monodominant bonebed: flood-induced	**Late Cretaceous**	Ryan et al. 2001
	Centrosaurus brinkmani	Dinosaur Provincial Park, AB	Monodominant bonebed	**Late Cretaceous**	Ryan and Russell 2005
	Styracosaurus albertensis	Dinosaur Provincial Park, AB	Monodominant bonebed	**Late Cretaceous**	
	Einiosaurus procurvicornis	Two Medicine Formation, Montana	Monodominant bonebed: drought assemblage	**Late Cretaceous**	Sampson 1995
	Pachyrhinosaurus n. sp.	Pipestone Creek, AB	Monodominant bonebed	**Late Cretaceous**	Fanti et al. 2015
	Medusaceratops			**Late Cretaceous**	
	Agujaceratops mariscalensis	Big Bend, Texas	Monodominant bonebed	**Late Cretaceous**	Lehman 1989
**Protoceratopsidae**	*Protoceratops andrewsi*	Tugriken Shireh, Mongolia	Clutch of hatchlings	**Late Cretaceous**	You et al. 2004
**Leptoceratopsidae**	*Prenoceratops peiganensis*	Two Medicine Formation, Montana	Monodominant bonebed	**Late Cretaceous**	Chinnery 2004
**Psittacosauridae**	*Psittacosaurus*		Parent protecting brood	**Late Cretaceous**	Meng et al. 2004
**Hadrosauridae**	*Maiasaura peeblesorum*	Montana	Monodominant bonebed	**Late Cretaceous**	Varricchio and Horner, 1993
	Prosaurolophus blackfeetensis	Judith River Fm., Montana	Monodominant bonebed: drought induced	**Late Cretaceous**	Rogers, 1990;
	Brachylophosaurus canadensis	Judith River Fm., Montana	Monodominant bonebed	**Late Cretaceous**	Prieto-Marquez, 2005
	Edmontosaurus annectens	South Dakota	Monodominant bonebed	**Late Cretaceous**	Derstler, 1994
	Edmontosaurus regalis	Edmonton, Alberta	Monodominant bonebed	**Late Cretaceous**	Bell and Campione, 2015
	Edmontosaurus sp. (Alaska)	Colville River, Alaska	Monodominant bonebed	**Late Cretaceous**	
	Hypacrosaurus altispinus			**Late Cretaceous**	Bramble and Currie, 2015
	Charonosaurus jiayinensis	Heilongjang, China		**Late Cretaceous**	Godefroit et al., 2000
	Bactrosaurus	Iren Dabasu, Mongolia			Godefroit, 1998
	Tenontosaurus tilletti		Monodominant bonebed	**Early Cretaceous**	Forster 1995
**Ankylosauridae**	*Pinacosaurus mephistocephalus*	Bayan Mandahu, Inner Mongolia	Family group overwhelmed by sandstorm	**Late Cretaceous**	Jerzykiewicz et al., 1993
	Pinacosaurus grangeri	Aleg Teg, Mongolia	Drought assemblage	**Late Cretaceous**	
**Thescelosauridae**	*Oryctodromeus cubicularis*	Blackleaf Formation, MT	Family group in burrow	**Late Cretaceous**	Varricchio et al., 2006

	Unnamed taxon	Proctor Lake, Texas			Winkler and Murry, 1989
**Ornithischia indet.**	*Laquintasaura venezuelae*	La Quinta Fm., Venezuela	Monodominant	**Early Jurassic**	Barrett et al. 2014
**Prosauropoda**	*Sellosaurus*				Hungerbühler, 1998
	Plateosaurus		Monodominant	**Late Triassic**	Sander, 1992
**Titanosauridae**	*Alamosaurus sanjuanensis*	Big Bend, Texas	Monodominant bonebed: drought assemblage?	**Late Cretaceous**	Fiorillo and Montgomery 2001
		Jones Ranch, Texas		**Early Cretaceous**	Winkler et al., 1997
**Diplodocidae**	*?Diplodocus*	Howe quarry	Monodominant bonebed	**Early Cretaceous**	
	Patagosaurus fariasi	Chubut, Argentina	Monodominant bonebed	**Jurassic**	Coria, 1994
**Coelophysoidea**	*Coelophysis bauri*	Ghost Ranch, New Mexico	Drought assemblage	**Late Triassic**	Colbert, 1989
	Coelophysis rhodesiensis	Rhodesia		**Early Jurassic**	Raath 1990
	Coelophysis kayentakatae, C. sp.	New Mexico		**Early Jurassic**	Rowe, 1989
**Monolophosaur-idae**	*Guanlong wucaii*	China	Monodominant	**Middle Jurassic**	Xu et al., 2006
**Carcharodonto-sauridae**	*Mapusaurus roseae*	Argentina	Monodominant	**Late Cretaceous**	Coria and Currie, 2006
**Allosauridae**	*Allosaurus fragilis*	Cleveland-Lloyd Quarry, UT	Drought assemblage	**Late Jurassic**	Madsen, 1976; Gates, 2005
**Tyrannosauridae**	*Daspletosaurus* sp.	Montana		**Late Cretaceous**	Currie et al., 2005
	Albertosaurus sarcophagus	Dry Island Buffalo Jump Provincial Park, Alberta	Monodominant bonebed. Pack?	**Late Cretaceous**	Currie, 1998
**Ornithomimidae**	*Sinornithomimus dongi*	Ulan Suhai, Inner Mongolia	Monospecific bonebed: social assemblage	**Late Cretaceous**	Kobayashi et al., 1999
**Therizinosauroidea**	*Falcarius utahensis*		Monodominant bonebed	**Early Cretaceous**	Kirkland et al. 2005
**Dromaeosauridae**	*Deinonychus antirrhopus*	Cloverly Formation, MT		**Early Cretaceous**	Ostrom, 1969; Maxwell and Ostrom, 1995
**Avimimidae**	*Avimimus. portentosus*	Nemegt Basin, Mongolia	Monodominant bonebed, flood assemblage?	**Late Cretaceous**	This paper

Figure S1: Graphs of skeletal representation of elements from the *Avimimus* bonebed, for a) an unfused skeleton; and b) a fused skeleton. Line represents average skeletal representation; values above the line indicate overrepresentation, values below indicate underrepresentation.

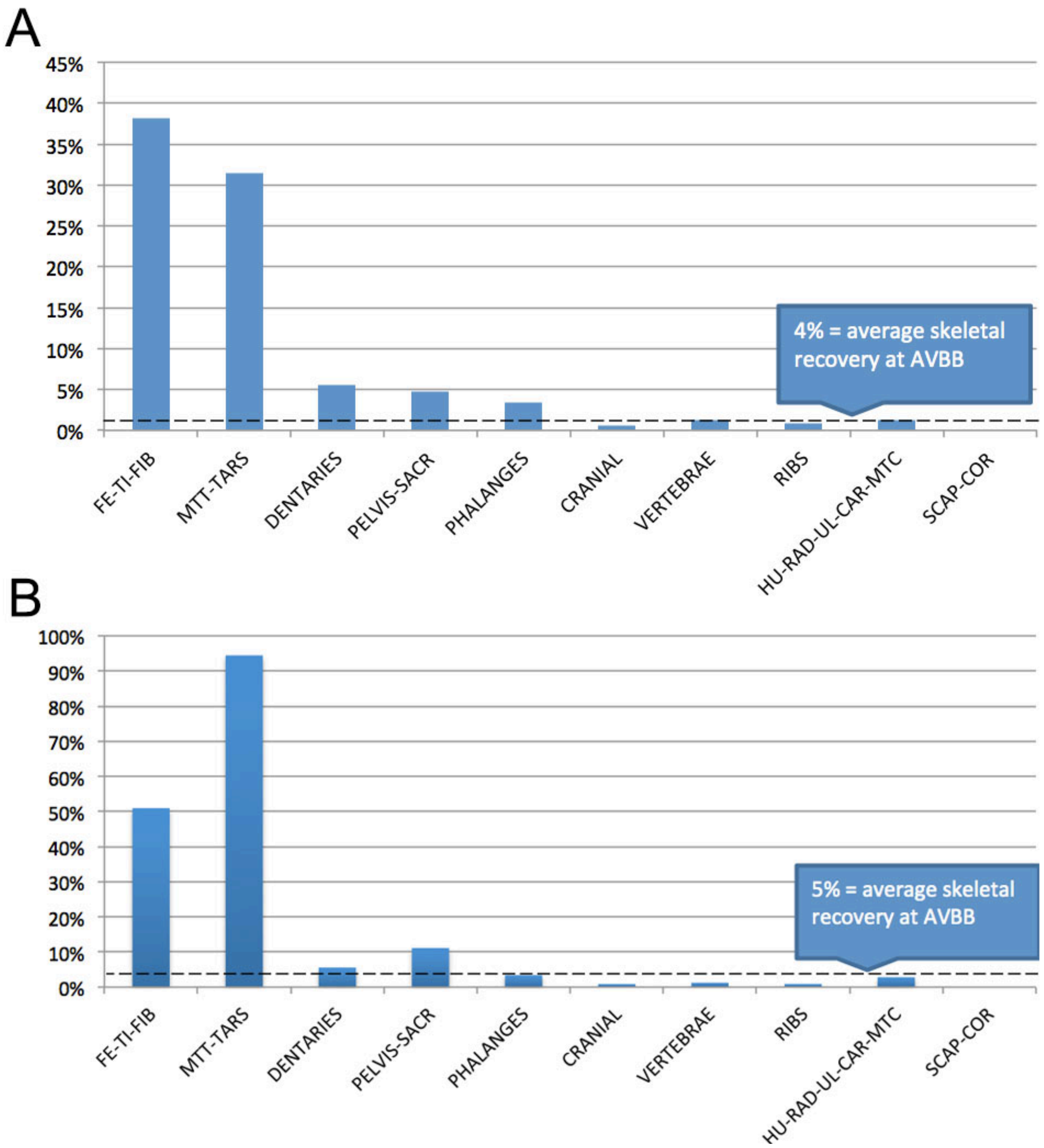

Figure S2: Articulated distal caudal vertebrae recovered from the *Avimimus* bonebed in 2016. Photograph by GFF (A) of caudal vertebrae as uncovered in the field. Outline (B) of caudal vertebrae, with each vertebra in a different colour. Abbreviations: **hum**, humerus.

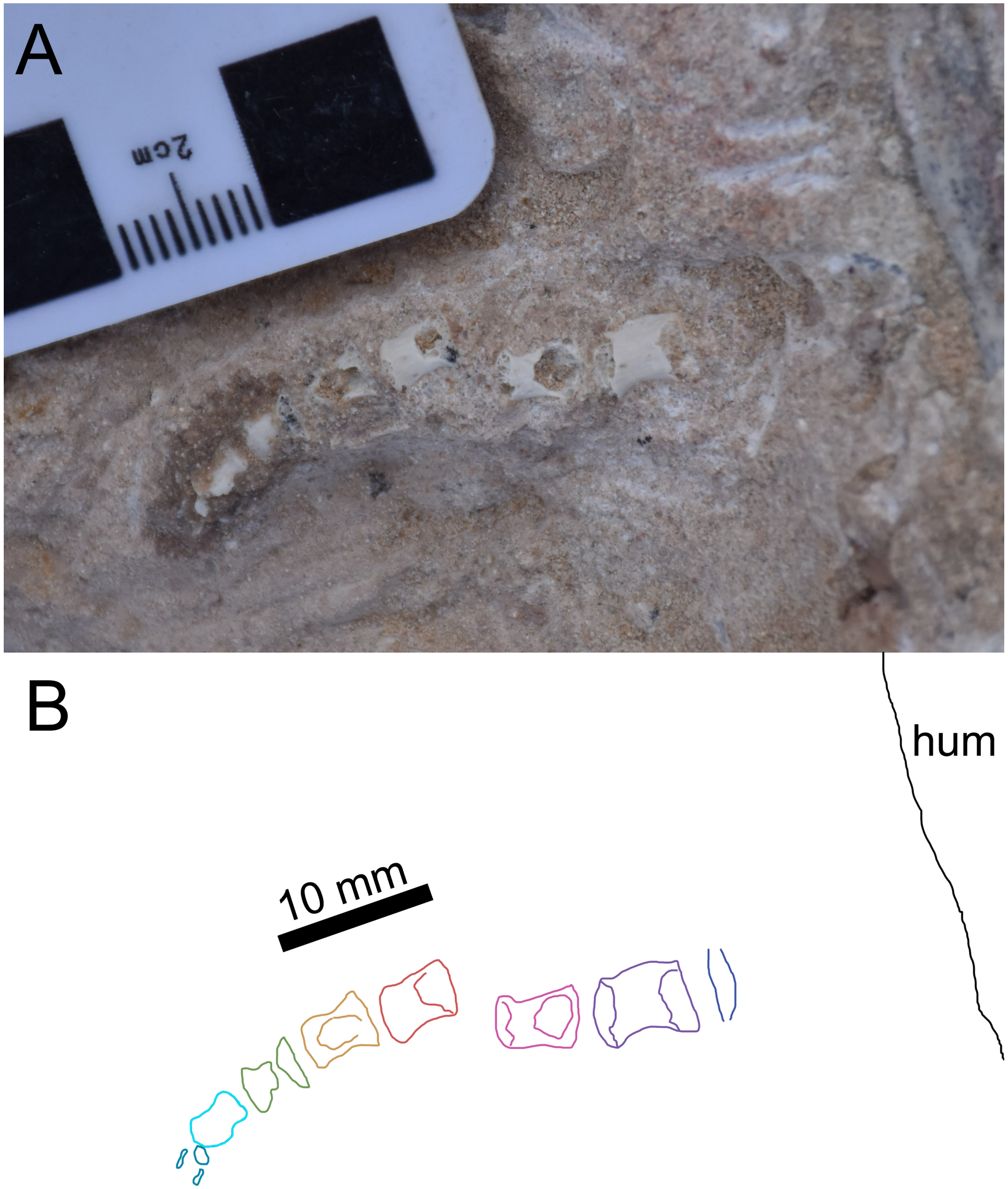

Figure S3: Articulated tibia and fibula recovered from the *Avimimus* bonebed. Note curved distal portion of fibula, and distal end of fibula fused to tibiatarsus (Arrow). Photograph by GFF.

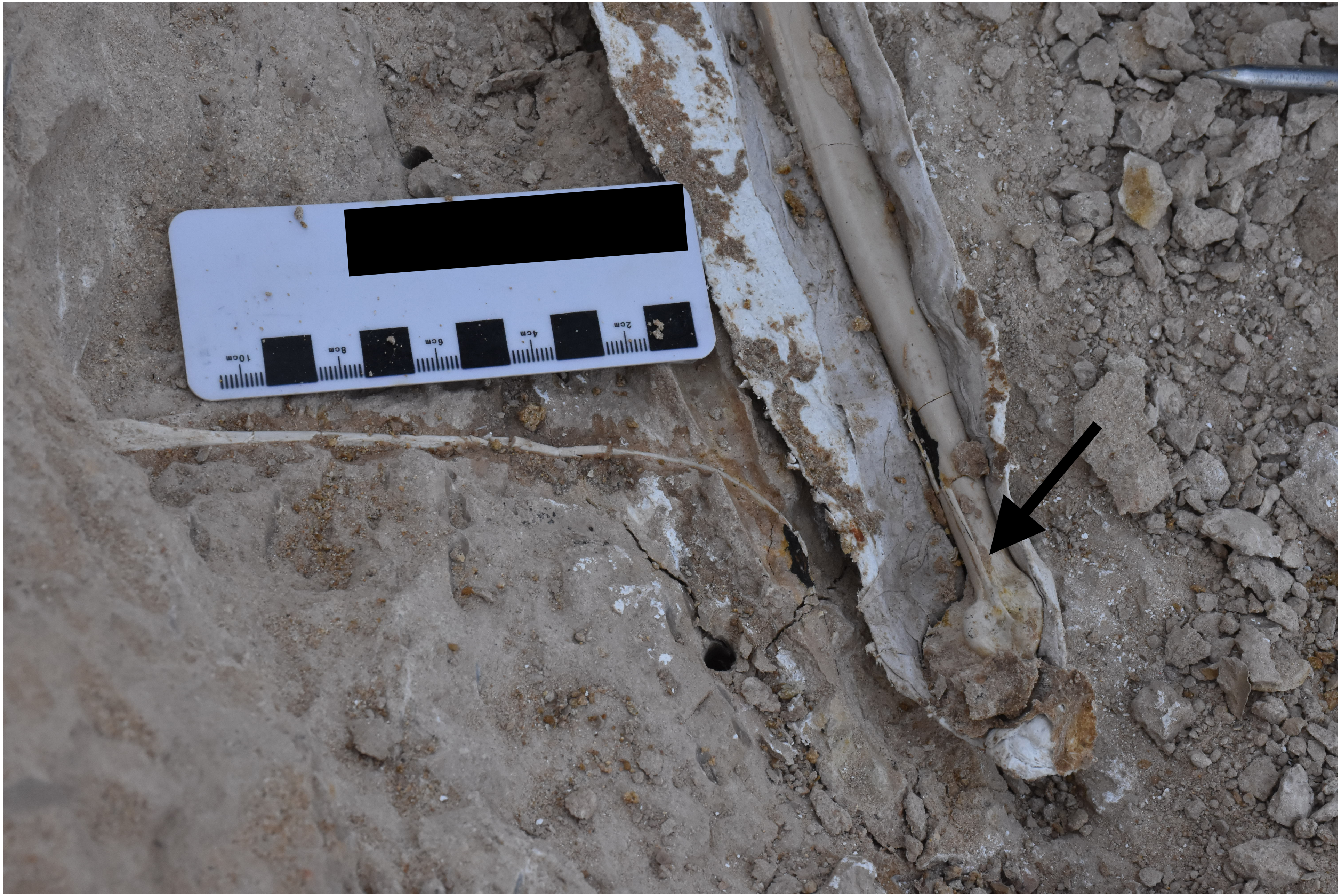

Literature Cited

Barrett, P.M., Butler, R.J., Mundil, R., Scheyer, T.M., Irmis, R.B., Sánchez-Villagra, M.R. 2014. A palaeoequatorial ornithischian and new constraints on early dinosaur diversification. Proceedings of the Royal Society B 281: 20141147.

Bell, P. R., and Campione, N. E. 2014. Taphonomy of the Danek Bonebed: a monodominant *Edmontosaurus* (Hadrosauridae) bonebed from the Horseshoe Canyon Formation, Alberta. Canadian Journal of Earth Sciences 51: 992-1006.

Bramble, K. K., and Currie, P. J. 2015. A juvenile *Hypacrosaurus altispinus* (Dinosauria: Hadrosauridae) bonebed from the Horseshoe Canyon Formation (Upper Cretaceous) of Alberta, Canada. Journal of Vertebrate Paleontology 35: 95A.

Chiba, K., Ryan, M. J., Braman, D. R., Eberth, D. A., Scott, E. E., Brown, C. M., Kobayashi, Y., and Evans, D. C. 2015. Taphonomy of a monodominant *Centrosaurus apertus* (Dinosauria: Ceratopsia) bonebed from the upper Oldman Formation of Southeastern Alberta. Palaios 30: 655-667.

Chinnery, B.J., 2004. Description of *Prenoceratops pieganensis* gen. et sp. nov. (Dinosauria: Neoceratopsia) from the Two Medicine Formation of Montan Journal of Vertebrate Paleontology 24, 572-590.

Colbert, E.H. 1989 The Triassic dinosaur *Coelophysis*. Museum of Northern Arizona Bulletin 57: 1-160.

Coria, R.A. 1994. On a monospecific assemblage of sauropod dinosaurs from Patagonia: implications for gregarious behaviour. Gaia 10: 209-213.

Coria, R.A., Currie, P.J., 2006. A new carcharodontosaurid (Dinosauria, Theropoda) from the Upper Cretaceous of Argentina. Geodiversitas 26, 71-118.

Currie, P. J. 1998. Possible evidence of gregarious behavior in tyrannosaurids. Gaia 15:271-277.

Currie, P.J., Trexler, D., Koppelhus, E.B., Wicks, K., Murphy, N. 2005. An unusual multi-individual tyrannosaurid bonebed in the Two Medicine Formatic (Late Cretaceous, Campanian) of Montana (USA). *In*: The Carnivorous Dinosaurs, *eds*. K. Carpenter. Indiana University Press, Indiana, pp. 313-32

Derstler, K. 1994. Dinosaurs of the Lance Formation in Eastern Wyoming. Forty-fourth Annual Field Conference, Wyoming Geological Association Guidebook, pp. 127-147.

Fanti, F., Currie, P.J., and Burns, M.E. 2015. Taphonomy, age, and paleoecological implication of a new *Pachyrhinosaurus* (Dinosauria: Ceratopsidae) bonebed from the Upper Cretaceous (Campanian) Wapiti Formation of Alberta, Canada. Canadian Journal of Earth Sciences 52: 250-260

Fiorillo, A.R., Montgomery, H., 2001. Depositional setting and paleoecological significance of a new sauropod bonebed in the Javelina Formation (Cretaceous) of Big Bend National Park, Texas. Journal of Vertebrate Paleontology Supplement 21: 49A

Forster, C.A. 1990. Evidence for juvenile groups in the ornithopod dinosaur *Tenontosaurus tilleti* Ostrom. Journal of Paleontology 64(1):164-165.

Gates, T.A., 2005. The Late Jurassic Cleveland-Lloyd Dinosaur Quarry as a drought-induced assemblage. Palaios 20, 363-375.

Godefroit, P., Dong, Z.-H., Bultynck, P., Li, H., and Feng, L. 1998. New *Bactrosaurus* (Dinosauria: Hadrosauroidea) material from Iren Dabasu (Inner Mongolia, P.R. China). Bulletin de l'Institut Royal Des Sciences Naturelles de Belgiques Science de la Terre 68: 3-70.

Godefroit, P., Zan, S., Jin, L. 2000. *Charonosaurus jiayinensis*, n.g., n.sp., a lambeosaurine dinosaur from the Late Maastrichtian of northeastern China. Comptes Rendus dle l'Académie de Sciences, Earth and Planetary Sciences 330: 875-882.

Hungerbühler, A. 1998. Taphonomy of the prosauropod dinosaur *Sellosaurus*, and its implications for carnivore faunas and feeding habits in the Late Triassic. Palaeogeography, Palaeoclimatology, Palaeoecology 143: 1-29.

Kirkland, J.I., 2005. A primitive therizinosauroid dinosaur from the Early Cretaceous of Utah. Nature 435, 84-87.

Kobayashi, Y., J.-C. Lu, Z.-M. Dong, R. Barsbold, Y. Azuma, and Y. Tomida. 1999. Herbivorous diet in an ornithomimid dinosaur. Nature 402:480-481.

Lehman, T. M., 1989. *Chasmosaurus mariscalensis*, sp. nov., a new ceratopsian dinosaur from Texas. Journal of Vertebrate Paleontology 9, 137-162.

Madsen, J. H. 1976. Allosaurus fragilis: a revised osteology. Utah Geological and Mineralogical Survey Bulletin 109:1-163.

Maxwell, W.D., Ostrom, J.H., 1995. Taphonomy and paleobiological implications of *Tenontosaurus-Deinonychus* associations. Journal of Vertebrate Paleontology 15, 707-712.

Meng, Q., Liu, J., Varricchio, D.J., Huang, T., Gao, C., 2004. Parental care in an ornithischian dinosaur. Nature 431, 145-146.

Ostrom, J.H. 1969. Osteology of *Deinonychus antirrhopus*, an unusual theropod from the Lower Cretaceous of Montana. Peabody Museum of Natural History Yale University Bulletin 30: 1-165.

Prieto-Marquez, A., 2005. New information on the cranium of *Brachylophosaurus canadensis* (Dinosauria, Hadrosauridae), with a revision of its phylogenetic position. Journal of Vertebrate Paleontology 25, 144-156.

Raath, M.A. 1990. Morphological variation in small theropods and its meaning in systematics: evidence from *Syntarsus rhodesiensis*. *In*: Carpenter K. & Currie P.J., (eds), Dinosaur Systematics, Approaches and Perspectives. New York, Cambridge University Press, 91-104

Rogers, R.R., 1990. Taphonomy of three dinosaur bonebeds in the Upper Cretaceous Two Medicine Formation of Northwestern Montana: evidence for drought-related mortality. Palaios 5, 394-413.

Ryan, M. J., and A. P. Russell. 2005. A new centrosaurine ceratopsid from the Oldman Formation of Alberta and its implications for centrosaurine taxonor and systematics. Canadian Journal of Earth Sciences 42:1369-1387.

Ryan, M. J., A. P. Russell, D. A. Eberth, and P. J. Currie. 2001. The taphonomy of a Centrosaurus (Ornithischia: Ceratopsidae) bonebed from the Dinosaur Park Formation (Upper Campanian), Alberta, Canada, with Comments on Cranial Ontogeny. Palaios 16:482-506.

Rowe, T. 1989. A new species of the theropod dinosaur *Syntarsus* from the Early Jurassic Kayenta Formation of Arizona. Journal of Vertebrate Paleontolo 9(2): 125-136.

Sampson, S.D., 1995. Two new horned dinosaurs from the Upper Cretaceous Two Medicine Formation of Montana; with a phylogenetic analysis of the Centrosaurinae (Ornithischia: Ceratopsidae). Journal of Vertebrate Paleontology 15, 743-760.

Sander, P. M. 1992. The Norian *Plateosaurus* bonebeds of central Europe and their taphonomy. Palaeogeography, Palaeoclimatology, Palaeoecology 93: 255-299.

Varricchio, D.J. and Horner, J.R. 1993. Hadrosaurid and lambeosaurid bone beds from the Upper Cretaceous Two Medicine Formation of Montana: taphonomic and biologic implications. Canadian Journal of Earth Sciences 30: 997-1006.

Varricchio, D. J., A. J. Martin, and Y. Katsura. 2006. First trace and body fossil evidence of a burrowing, denning dinosaur. Proceedings of the Royal Society B:1-7.

Winkler, D.A., and Murry, P.A. Paleoecology and hypsilophodontid behaviour at the Proctor Lake dinosaur locality (Early Cretaceous), Texas. *In*: Farlow, J.O. ed. Paleobiology of the dinosaurs: Boulder, Colorado, Geological Society of America Special Paper 238.

Winkler, D.A., Murry, P.A., and Jacobs, L.L. 1997. A new species of *Tenontosaurus* (Dinosauria: Ornithopoda) from the Early Cretaceous of Texas. Journ of Vertebrate Paleontology 17(2): 330-348.

Xu, X., Clark, J.M., Forster, C.A., Norell, M.A., Erickson, G.M., Eberth, D.A., Jia, C., and Zhao, Q. 2006. A basal tyrannosauroid dinosaur from the Late Jurassic of China. Nature 439(9): 715-718.

You, H., Dodson, P. 2004. Basal Ceratopsia. In Weishampel, D.B., Dodson, P. and Osmolska, H. (Eds). The Dinosauria. University of California Press, Berkeley, pp 478-493.
